# Assessing the relationship between the rumen microbiota and feed efficiency in Nellore steers

**DOI:** 10.1186/s40104-021-00599-7

**Published:** 2021-07-15

**Authors:** Déborah Romaskevis Gomes Lopes, Márcio de Souza Duarte, Alex J. La Reau, Ibrahim Zuniga Chaves, Tiago Antônio de Oliveira Mendes, Edenio Detmann, Cláudia Braga Pereira Bento, Maria Eugênia Zerlotti Mercadante, Sarah Figueiredo Martins Bonilha, Garret Suen, Hilário Cuquetto Mantovani

**Affiliations:** 1 Departamento de Microbiologia, Universidade Federal de Viçosa, Viçosa, MG 36570-900 Brazil; 2 Departamento de Zootecnia, Universidade Federal de Viçosa, Viçosa, Minas Gerais Brazil; 3 Department of Bacteriology, University of Wisconsin-Madison, Madison, WI USA; 4 Departamento de Bioquímia e Biologia Molecular, Universidade Federal de Viçosa, Viçosa, Minas Gerais Brazil; 5 Instituto de Zootecnia, Centro APTA Bovinos de Corte, Sertãozinho, São Paulo, Brazil

**Keywords:** Beef cattle, ITS1 region, Next-generation sequencing, RFI, Rumen microbiota, 16S rRNA gene

## Abstract

**Background:**

Ruminants rely upon a complex community of microbes in their rumen to convert host-indigestible feed into nutrients. However, little is known about the association between the rumen microbiota and feed efficiency traits in Nellore (*Bos indicus*) cattle, a breed of major economic importance to the global beef market. Here, we compare the composition of the bacterial, archaeal and fungal communities in the rumen of Nellore steers with high and low feed efficiency (FE) phenotypes, as measured by residual feed intake (RFI).

**Results:**

The Firmicutes to Bacteroidetes ratio was significantly higher (*P* < 0.05) in positive-RFI steers (p-RFI, low feed efficiency) than in negative-RFI (n-RFI, high feed efficiency) steers. The differences in bacterial composition from steers with high and low FE were mainly associated with members of the families Lachnospiraceae, Ruminococcaceae and Christensenellaceae, as well as the genus *Prevotella*. Archaeal community richness was lower (*P* < 0.05) in p-RFI than in n-RFI steers and the genus *Methanobrevibacter* was either increased or exclusive of p-RFI steers. The fungal genus *Buwchfawromyces* was more abundant in the rumen solid fraction of n-RFI steers (*P* < 0.05) and a highly abundant OTU belonging to the genus *Piromyces* was also increased in the rumen microbiota of high-efficiency steers. However, analysis of rumen fermentation variables and functional predictions indicated similar metabolic outputs for the microbiota of distinct FE groups.

**Conclusions:**

Our results demonstrate that differences in the ruminal microbiota of high and low FE Nellore steers comprise specific taxa from the bacterial, archaeal and fungal communities. Biomarker OTUs belonging to the genus *Piromyces* were identified in animals showing high feed efficiency, whereas among archaea, *Methanobrevibacter* was associated with steers classified as p-RFI. The identification of specific RFI-associated microorganisms in Nellore steers could guide further studies targeting the isolation and functional characterization of rumen microbes potentially important for the energy-harvesting efficiency of ruminants.

**Supplementary Information:**

The online version contains supplementary material available at 10.1186/s40104-021-00599-7.

## Background

With the projected growth of the world’s population in the next decades, it is expected that meat production will have to double to meet the global demand for animal protein [1]. Ruminants, which have a major role in ensuring sustainable food security, consume about 30% of the crops cultivated on Earth and occupy another 30% of the planet’s surface [2, 3]. Therefore, expanding herd size and land use to meet projected food demands is undesirable due to the environmental impacts associated with livestock production [3]. In this context, there is an increasing interest in the use of feed efficiency (FE) indexes/markers in animal breeding programs to select for animals that produce more meat or milk while consuming less feed [4]. In beef cattle, residual feed intake (RFI), which is the difference between observed dry matter intake (DMI) and expected DMI based on animal development metrics [5], has been widely used to calculate the FE of growing cattle. The expected DMI is determined from linear regression of measured feed intake versus developmental measures (such as mean daily weight gain) for a group of animals on the same diet. However, previous studies demonstrated that RFI has low to moderate heritability in cattle (h^2^ = 0.25, h^2^ = 0.39), which suggests that FE is also affected by non-genetic factors [6, 7].

The feed conversion in ruminants depends both on the ability of the rumen microbiota to ferment the components of their diet to produce volatile organic acids, and the ability of these animals to absorb and utilize these nutrients (genetic potential). Therefore, ruminal microorganisms play a fundamental role in providing energy to their hosts [8]. Although feed efficiency is considered a multifactorial and complex trait in cattle involving multiple biological processes connected to the host [2, 9], several published studies provide evidence for associations between the rumen microbiome and RFI phenotypes in both dairy cows [2, 10–12] and beef cattle [13–19]. It has been proposed that cows that are more efficient host a microbiome with lower richness and diversity, but produce higher abundances of important metabolites for the host [2]. Most importantly, the functional analysis of the rumen microbiota indicates that differences in animals of high and low FE may be related to the genes involved in the digestion of fibrous and non-fibrous carbohydrates, synthesis of fatty acids and proteins, pathways for conservation of metabolic energy (e.g., ATP production), and methane production [11].

However, the ecological understanding of the rumen microbiome in *Bos indicus* cattle is still limited, especially the compositional dynamics of the rumen microbiome and its association with productivity traits of Nellore cattle [20, 21], which accounts for more than 90% of Brazilian beef cattle herds and represents one of the most abundant sources of meat worldwide [1, 22]. Also, linear regression models predicting rumen bacterial features affecting feed efficiency indicated that the rumen microbiome likely explains approximately 20% of the variation in feed efficiency traits in beef steers [19]. Therefore, research on the association between the rumen microbiota and feed efficiency traits (e.g. RFI) in Nellore (*Bos indicus*) cattle is needed to unveil the potential rumen microbial signatures in animals that are more efficient at feed utilization.

To address this, we examined differences in the composition of the ruminal microbiota (Bacteria, Archaea, and Fungi) in the solid and liquid rumen contents of Nellore cattle showing high and low FE phenotypes (negative and positive RFI, respectively). This exploratory study aimed to determine if specific rumen microbes contribute to the feed efficiency phenotype in Nellore steers, a feature that could be relevant in evaluating the phenotypic variation of FE in *Bos indicus* cattle. The present study also provides the first systematic account of the bacterial, archaeal, and fungal communities colonizing the rumen of Nellore cattle showing distinct FE phenotypes.

## Materials and methods

### Animals, diets and sampling

A cohort of 129 young Nellore bulls (7 months of age, 239 ± 30.1 kg of initial body weight, BW) were fed a diet formulated to meet the requirements for 1 kg/d of BW gain during a growth period of 98 d [20, 23, 24]. The diet was formulated without ionophore supplementation and the composition was: 615 g/kg corn silage, 33 g/kg *Brachiaria* hay, 167 g/kg dry ground corn, 163 g/kg soybean meal, 3.6 g/kg urea, 0.4 g/kg ammonium sulfate, and 18 g/kg mineral mixture (on a dry matter basis). All animals had *ad libitum* access to water throughout the experiment. Before the beginning of the test period, all bulls were weighed, dewormed, vaccinated, and numbered with individual tags [20, 23, 24].

Cattle were fed using a GrowSafe automated feeding system (GrowSafe Systems Ltd., Airdrie, Canada). The RFI (kg/d) was calculated during the growth period as the error term of the following equation: DMI = β_0_ + β_P_ × BW_0.75_ + β_G_ × ADG + ε(RFI), where DMI is the dry matter intake observed during the test period, β_0_ is the intercept of the equation, BW_0.75_ is the mid-test metabolic live weight, ADG is the average daily gain in weight during the test, and β_P_ and β_G_ are the regression coefficients of BW_0.75_ and ADG, respectively. ADG was estimated by the linear regression coefficient of live weight as a function of the number of days in the test. Mid-test metabolic live weight was calculated by the equation BW_0.75_ = [α + β × (DIT/2)]_0.75_, where α is the intercept of the regression equation corresponding to the initial live weight, β is the linear regression coefficient corresponding to the average daily gain, and DIT is the days in test [24].

From the original group of 126 individuals, a cohort of 27 steers (12 with negative RFI and 15 with positive RFI) were selected based on a stratified sampling method for the finishing period to increase the statistical power [25]. Individuals sampled in the first stage represented the 20% most efficient and the 20% least efficient steers of the cohort (*P* < 0.0001, Supplementary Table S1), followed by random sampling of an equal number of individuals (*n* = 9) belonging to the non-extreme RFI group. The RFI values were significantly different (*P* < 0.05) between animals with negative (− 0.93 ± 0.17) or positive (0.87 ± 0.14) RFI and the steers were separated into two FE groups: negative-RFI (n-RFI, more efficient steers) and positive-RFI (p-RFI, less efficient steers), respectively. The steers averaged 22.5 ± 0.8 mo of age and 401 ± 42 kg of BW, were confined in individual pens (4 m × 2 m) equipped with GrowSafe automated feeding systems (GrowSafe Systems Ltd., Airdrie, Alberta, Canada) and had *ad libitum* access to the diet and water. The steers were adapted to the diets, facilities, and management for 22 d followed by a period of 103 d where they received the finishing diet, formulated to meet the requirements of 1.3 kg of daily gain with a target finishing weight of at least 550 kg [20, 23, 24]. The finishing diet was composed of 333 g/kg corn silage, 17 g/kg *Brachiaria* hay, 465 g/kg dry ground corn, 163 g/kg soybean meal, 6 g/kg urea, 4 g/kg ammonium sulfate, and 13 g/kg mineral mixture (dry matter basis).

After slaughter, the ruminal contents were collected and filtered through four layers of cheese cloth to separate the liquid and solid fractions. Ruminal pH was measured in the liquid fraction using a portable pH meter (pH meter HI9124, Hanna Instruments, Woonsocket, Rhode Island, EUA). Aliquots from each fraction were stored in plastic containers at − 20 °C for further analysis.

### Ammonia and volatile fatty acids measurement

Ammonia concentration was determined according to Chaney and Marbach [26]. Absorbance was measured at 630 nm in a Spectronic 20D spectrophotometer (Thermo Fisher Scientific, Madison, WI, USA) and ammonium chloride (NH_4_Cl) was used as the standard.

Quantification of organic acids was performed using cell-free supernatants of the rumen fluid samples (2.0 mL) prepared as previously described [20]. The VFAs were determined in a Dionex Ultimate 3000 Dual detector HPLC (Dionex Corporation, Sunnyvale, CA, USA) equipped with a Shodex RI-101 refractive index (RI) detector and a Phenomenex Rezex ROA column (300 × 7.8 mm) (Phenomenex Inc. Torrance, CA, USA). Analyses were performed isocratically under the following conditions: mobile phase H_2_SO_4_ 5 mmol/L, flow rate 0.7 mL/min, column temperature 45 °C and injection volume 20 μL. Stock solutions of the external standards were prepared as previously described [20].

### DNA extraction and sequencing

Total genomic DNA was extracted separately from the ruminal solids and liquids (*n* = 54) of each animal following a mechanical disruption and phenol/chloroform extraction protocol as described by Stevenson and Weimer [27]. Extracted genomic DNA was quantified using a Nanodrop spectrophotometer (Thermo Scientific, Wilmington, DE). The V4 hypervariable region of the bacterial 16S rRNA gene was amplified using universal primers (F- GTGCCAGCMGCCGCGGTAA; R- GGACTACHVGGGTWTCTAAT) as described by Kozich et al. [28]. Furthermore, the archaeal 16S rRNA was amplified using primers for the V6-V8 hypervariable region (Ar915aF-AGGAATTGGCGGGGGAGCAC, Ar1386R-GCGGTGTGTGCAAGGAGC) and fungal sequences were amplified with primers for the internal transcribed spacer 1 (ITS1) region (MN100F-TCCTACCCTTTGTGAATTTG, MNGM2-CTGCGTTCTTCATCGTTGCG) as described by Kittelmann et al. [29]. For Bacteria, PCR reactions consisted of 50 ng template DNA, 0.4 μmol/L of each primer, 1 × Kapa Hifi HotStart Ready Mix (KAPA Biosystems, Cape Town, South Africa), and water to 25 μL. For Archaea and Fungi, DNA input was increased to 100 ng and primers to 1.6 μmol/L each. PCR was performed at 95 °C for 3 min, 95 °C for 30 s, 55 °C for 30 s, 72 °C for 30 s (25 cycles for Bacteria and 35 cycles for Archaea and Fungi), and a final extension step at 72 °C for 5 min [20, 30, 31]. PCR products were purified using a PureLink Pro 96 PCR Purification Kit (Invitrogen, Carlsbad, CA, EUA) and a second PCR was performed to attach both the Illumina sequencing adapters (F - AATGATACGGCGACCACCGAGATCTACAC; R - CAAGCAGAAGACGGCATACGAGAT) and unique barcodes to facilitate multiplexing. The second PCR reaction was similar to that for the bacterial V4 regions, except that 5 μL of non-quantified PCR product was used as template DNA and 8 cycles were performed [20, 30, 31]. PCR products were recovered by gel extraction in AquaPōr low-melt agarose (National Diagnostics, Atlanta, GA) using a Zymoclean Gel DNA Recovery Kit (Zymo Research, Irvine, CA, USA). Purified DNA was quantified using a Qubit fluorometer (Invitrogen) and equimolarly pooled to create a single sample at 1 × 10^9^ ng per μL [20, 30, 31]. Sequencing was performed using an Illumina MiSeq Reagent Kit v2 kit for Bacteria (2 × 250 bp) and an Illumina MiSeq Reagent Kit v3 for Achaea and Fungi (2 × 300 pb) on an Illumina MiSeq (Illumina, Inc., San Diego, CA, USA) following manufacturer’s guidelines. All DNA sequences have been deposited into the NCBI’s Sequence Read Archive (SRA) under BioProject accession number PRJNA512996.

### Sequence analysis

Bacterial, Archaeal and Fungal sequences were processed separately using mothur (v 1.43.0) [32]. Any sequences shorter than 200 bp or longer than 500 bp for Bacteria and shorter than 200 bp or longer than 600 bp for Archaea and Fungi were removed. The V4 and V6-V8 sequences of bacterial and archaeal 16S rRNA gene, respectively, were aligned using the SILVA 16S rRNA gene reference database (release 138) [33]. The fungal ITS1 sequences were aligned using the UNITE database version v8.2 (2020-02-04) [34] and sequences that did not align to the correct location were removed [20]. To reduce computational time and account for sequencing error, identical sequences were grouped using the *unique.seqs* command in mothur and sequences that were different by two or fewer base pairs were considered the same and grouped using *pre.cluster* command. Chimeric sequences [35] and singletons (sequences that occur once in the entire dataset) were removed as they provide little usable data and are often the result of sequencing error. All sequences were grouped into operational taxonomic units (OTUs) by uncorrected pairwise distance clustering using either the furthest neighbor (Bacteria and Archaea) or average neighbor (Fungi) method with a similarity cut-off of 97%. The bacterial and archaeal OTUs were classified using the SILVA database (release 138) and fungal OTUs were classified using the UNITE database v8.2 (2020-02-04), with a bootstrap cut-off of 80. Sample coverage was assessed using Good’s coverage and the relative abundance (reads/total reads in a sample) of OTUs was determined. The OTU tables were normalized using the *normalize.shared* command and method = totalgroup (relative abundance x lowest number of sequences per sample). Normalized OTU tables were used to determine alpha diversity indices (Chao1, Shannon’s and Simpson’s) and for beta diversity analyses.

### Statistical analysis

Differences in the alpha diversity indices and the Firmicutes to Bacteroidetes ratio, as well as the concentration of ammonia and organic acids of rumen samples, according to each FE group, were assessed by *t*-test as performed in Minitab 17.1.0 (Minitab, Inc., Pennsylvania, USA) with *P* < 0.05 being considered significant.

Clustering of the steers according to the composition of rumen contents was visualized using Non-metric multidimensional scaling (NMDS) plots of the Bray-Curtis dissimilarity metric (beta diversity index), and non-parametric analysis of similarities (ANOSIM, number of permutation = 10,000) were performed using the Past software package [36], with *P-*values < 0.001 being considered significant. Differences in the taxonomic composition according to FE group were assessed by White’s non-parametric *t*-test using the Statistical Analysis of Taxonomic and Functional Profiles – STAMP v 2.1.3 software [37], with *P-*values < 0.05 being considered significant.

For determining biomarkers, the Linear discriminant analysis Effect Size – LEfSe [38] at the OTU level was performed using Galaxy Version 1.0 (https://huttenhower.sph.harvard.edu/galaxy/). *P-*values < 0.05 for the factorial Kruskal-Wallis test and the pairwise Wilcoxon test were considered significant. The threshold for the logarithmic linear discrimination analysis (LDA) scores of discriminative features was 2, and the strategy for multi-class analysis was “all-against-all”.

Prediction of metabolic functions from the ruminal bacterial microbiota was performed using the CowPI/BeefPIE tool [39] that is used with an installation of the PICRUSt software package [40]. This analysis was performed in the Galaxy platform Version 0.1) (https://sharegalaxy.ibers.aber.ac.uk/?tool_id=beefpie_rstep&version=0.1&__identifer=zb2ieybu7r) using normalized OTU tables of the bacterial composition for each steer and in addition to nucleotide sequence representatives of each OTU. Comparison of the relative abundances of the predicted metabolic categories according to FE group was assessed by White’s non-parametric t-test using STAMP v 2.1.3 [37].

The correlation network of liquid and solid bacterial OTUs and ruminal fermentation variables was determined based on sequences that were detected in at least 50% of the steers classified in each FE group (minimum of 7 steers to p-RFI group and 6 steers to n-RFI group) to avoid spurious findings due to between-animal variability. This criterion was based on previous observations indicating that different species of ruminal bacteria that exhibit a heritable component have a high presence (≥ 50%) across animals [41]. Spearman’s rank correlation was calculated in R (v 3.4.1, corrr package) to assess the relationship of ammonia concentration, proportions of VFAs (acetic, succinic, propionic, valeric, isovaleric, isobutyric and butyric acid), total VFA concentration and acetate-to-propionate ratio against the relative abundance of bacterial OTUs. Significant correlations (*P* < 0.05) were visualized as a network in Cytoscape v 3.2.1 [42]. The networks were built using sheets with shared and exclusives correlations of each FE group.

## Results

### Sequencing

In total, 3,201,058, 1,202,160 and 2,415,859 raw sequences for Bacteria, Archaea and Fungi, respectively, were generated. After trimming, quality filtering and removal of chimeras, a total of 1,727,202 high-quality bacterial, 251,013 archaeal, and 1,044,332 fungal sequences were obtained. The Good’s coverage before and after normalization was > 95% for the bacterial community, > 99% for the archaeal community and > 94% for the fungal community, indicating sufficient coverage for comparisons of sequence abundance between samples and analysis of community shifts. The summary or sequence counts and OTUs that passed the quality filter, clean up and normalization are presented in Supplementary Table S2.

### Specific bacterial OTUs are associated with the p-RFI and n-RFI phenotypes

**Fig. 1:**
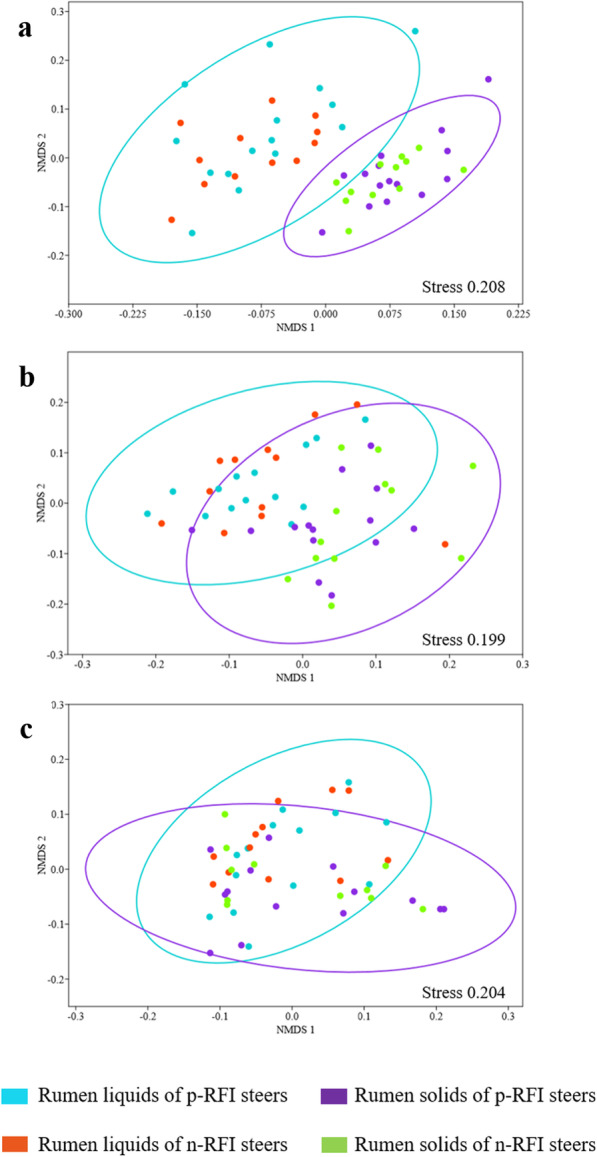
Non-metric multidimensional scaling (NMDS) plots of the Bray-Curtis dissimilarity index for bacterial (**a**) archaeal (**b**) and fungal (**c**) communities in the rumen of Nellore steers. Individual points represent a rumen sample and colors represent ruminal fractions and RFI groups

Alpha diversity was measured using Chao1, Simpson’s, and Shannon’s diversity indices and demonstrated that the bacterial community of Nellore steers did not vary between RFI groups (t-test, *P* > 0.05), regardless of the ruminal phase (Table 1). Beta diversity analysis showed that the Bray-Curtis dissimilarities of the bacterial communities differed according to the ruminal phase (solid or liquid fraction) (ANOSIM, *P* < 0.001), whereas the dissimilarity in bacterial communities between p-RFI and n-RFI did not vary significantly in the ruminal contents (Fig. 1a). These results demonstrate that microbial communities in the solid and liquid rumen phases are distinct and reinforce the need for separate analyses to evaluate the contributions of the feed-attached and planktonic ruminal microbiota to FE.

**Table 1:** Alpha diversity of microbial communities in the rumen of steers according to RFI group^a^

	Liquid			Solid		
	p-RFI	n-RFI	***P***-value	p-RFI	n-RFI	***P***-value
Bacteria						
Chao	1730 ± 301	1750 ± 281	0.863	1442 ± 388	1564 ± 379	0.417
Shannon	5.35 ± 0.34	5.44 ± 0.21	0.404	5.28 ± 0.27	5.32 ± 0.20	0.690
Simpson	0.018 ± 0.008	0.015 ± 0.007	0.345	0.017 ± 0.006	0.016 ± 0.006	0.903
Archaea						
Chao	37.57 ± 9.36	48.11 ± 12.93	< 0.05	34.87 ± 6.87	42.26 ± 9.27	< 0.05
Shannon	1.86 ± 0.31	1.72 ± 0.34	0.292	1.81 ± 0.24	1.78 ± 0.33	0.898
Simpson	0.26 ± 0.12	0.3 ± 0.15	0.488	0.27 ± 0.09	0.28 ± 0.13	0.980
Fungi						
Chao	289 ± 390	151 ± 71	0.199	128 ± 78	143 ± 71	0.602
Shannon	2.33 ± 0.42	2.27 ± 0.38	0.671	2.03 ± 0.65	2.18 ± 0.38	0.461
Simpson	0.20 ± 0.10	0.20 ± 0.09	0.999	0.29 ± 0.20	0.24 ± 0.13	0.452

^a^Values of alpha diversity represent the mean ± standard deviation

Taxonomic analysis of the ruminal bacterial community of the liquids fraction revealed 3,672 unique OTUs (mean 1,073 ± SD 108 per sample after normalization) that were assigned to 20 phyla, 38 classes, 67 orders, 110 families, and 237 genera. The rumen liquid (RL) bacterial community was dominated by the phyla Firmicutes (55.74 ± 2.17%), Bacteroidetes (20.53 ± 1.5%) (Supplementary Fig. S1). The most abundant families included the Ruminococcaceae (21.23 ± 1.02%), Lachnospiraceae (15.87 ± 1.6%), Prevotellaceae (10.16 ± 1.26%), and Christensenellaceae (5.81 ± 1.03%) (Supplementary Fig. S2). In the rumen solids (RS), 3342 unique bacterial OTUs (mean of 962 ± 132 OTUs per sample after normalization) were assigned to 18 phyla, 32 classes, 62 orders, 97 families, and 209 genera. Members of the phyla Firmicutes (59.17 ± 1.65%) and Bacteroidetes (20.58 ± 1.60%) were also dominant in the RS bacterial community (Supplementary Fig. S1), while abundant families included the Ruminococcaceae (20.57 ± 1.91%), Lachnospiraceae (20.18 ± 1.30%), Prevotellaceae (10.17 ± 1.20%) and Christensenellaceae (6.65 ± 0.94%) (Supplementary Fig. S2).

**Fig. 2:**
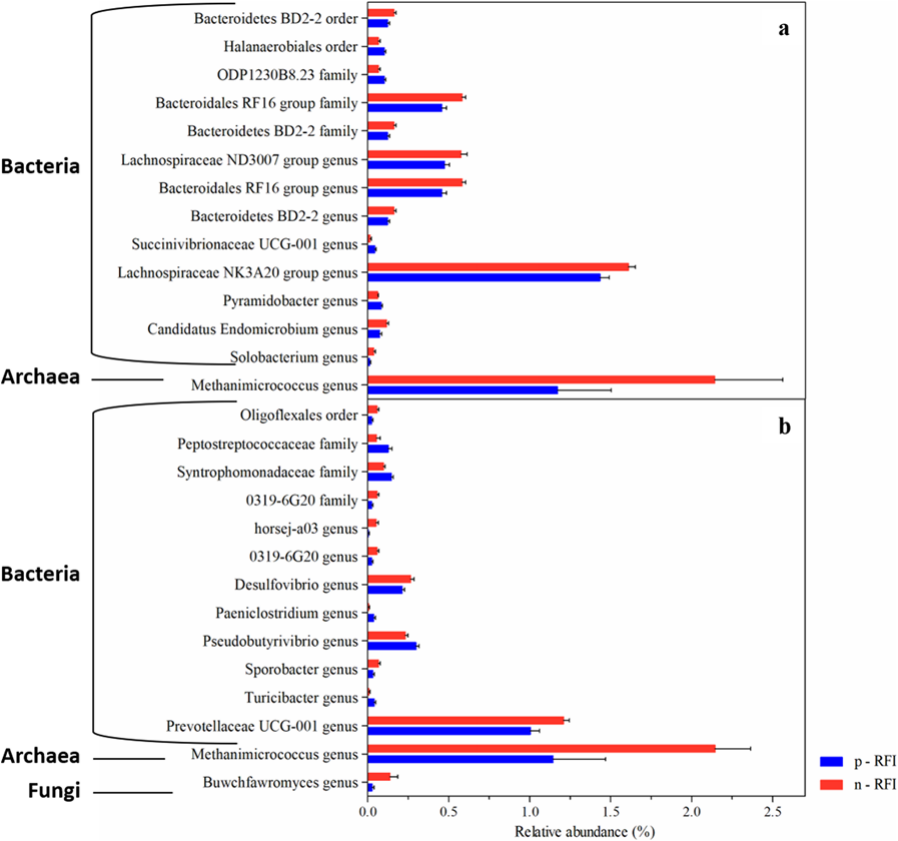
Taxonomic groups showing significant differences (White’s non-parametric t-test, *P* < 0.05) in relative abundance in liquid (**a**) and solid (**b**) ruminal fractions between p-RFI and n-RFI steers. The blue bars represent the p-RFI group and the red bars represent n-RFI group. Data are expressed as relative abundance mean and the standard error

The bacterial taxonomic composition of the animals was similar, however, the abundance of some taxonomic categories varied according to the FE. More efficient animals (n-RFI) had higher abundances (White’s non-parametric t-test, *P* < 0.05) of order Anaeroplasmatales and Lineage I (class Elusimicrobia), family ODP1230B8–23 (order Halanaerobiales), and genera Lachnospiraceae NK3A20 group, Bacteroidales RF16 group, Lachnospiraceae ND3007 group and Bacteroidetes BD2–2 in the RL. Also, the genera *Desulfovibrio*, horsej-a03 (family Oligosphaeraceae), and the Prevotellaceae UCG-001 were more abundant in the RS of n-RFI steers, when compared to the RS of p-RFI steers (Fig. 2).

**Fig. 3:**
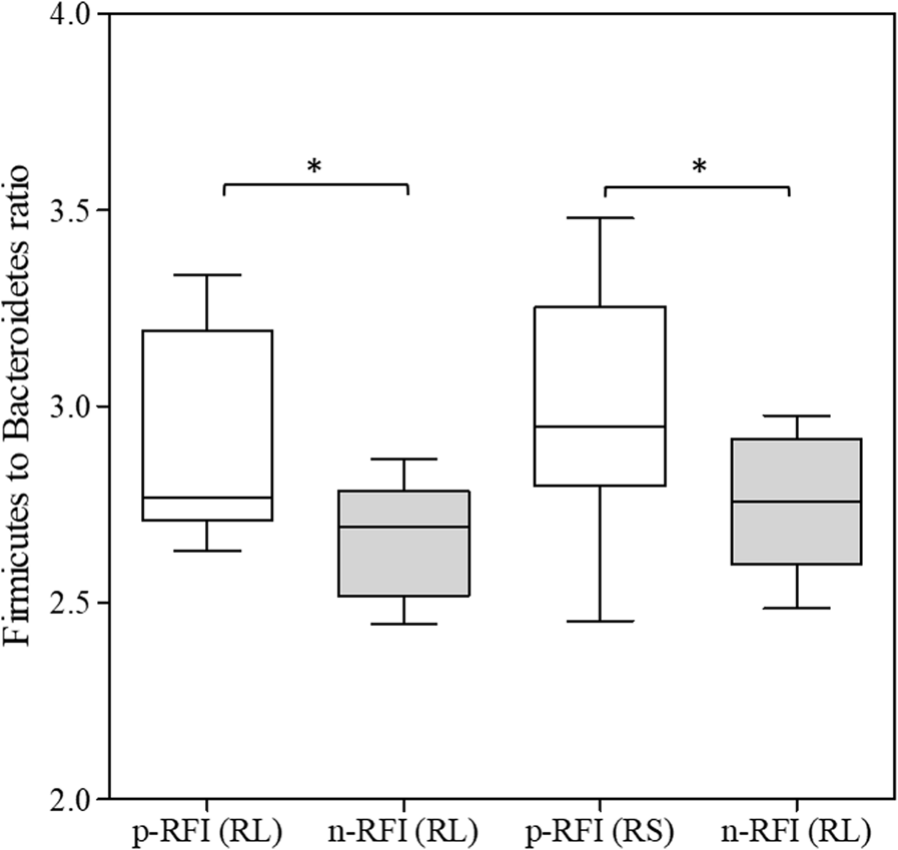
The *Firmicutes* to *Bacteroidetes* ratio in liquid (RL) and solid (RS) ruminal phase, according to the RFI group. White bars represent the p-RFI steers and grey bars represent the n-RFI steers. (*) Significantly different (*P* < 0.05) by the *t*-test

The Firmicutes to Bacteroidetes (F/B) ratio, an index previously reported as being associated with dysbiosis or differences in phenotypes in humans, rodents, and ruminants, was significantly higher (t-test, *P* < 0.05) in p-RFI steers than in n-RFI steers (Fig. 3). In the RL fraction, the average F/B ratio was 2.90 ± 0.07 for the p-RFI steers and 2.67 ± 0.04 for the n-RFI steers. In the RS fraction, the average ratio was 2.99 ± 0.08 for the p-RFI and 2.75 ± 0.05 for the n-RFI.

**Fig. 4:**
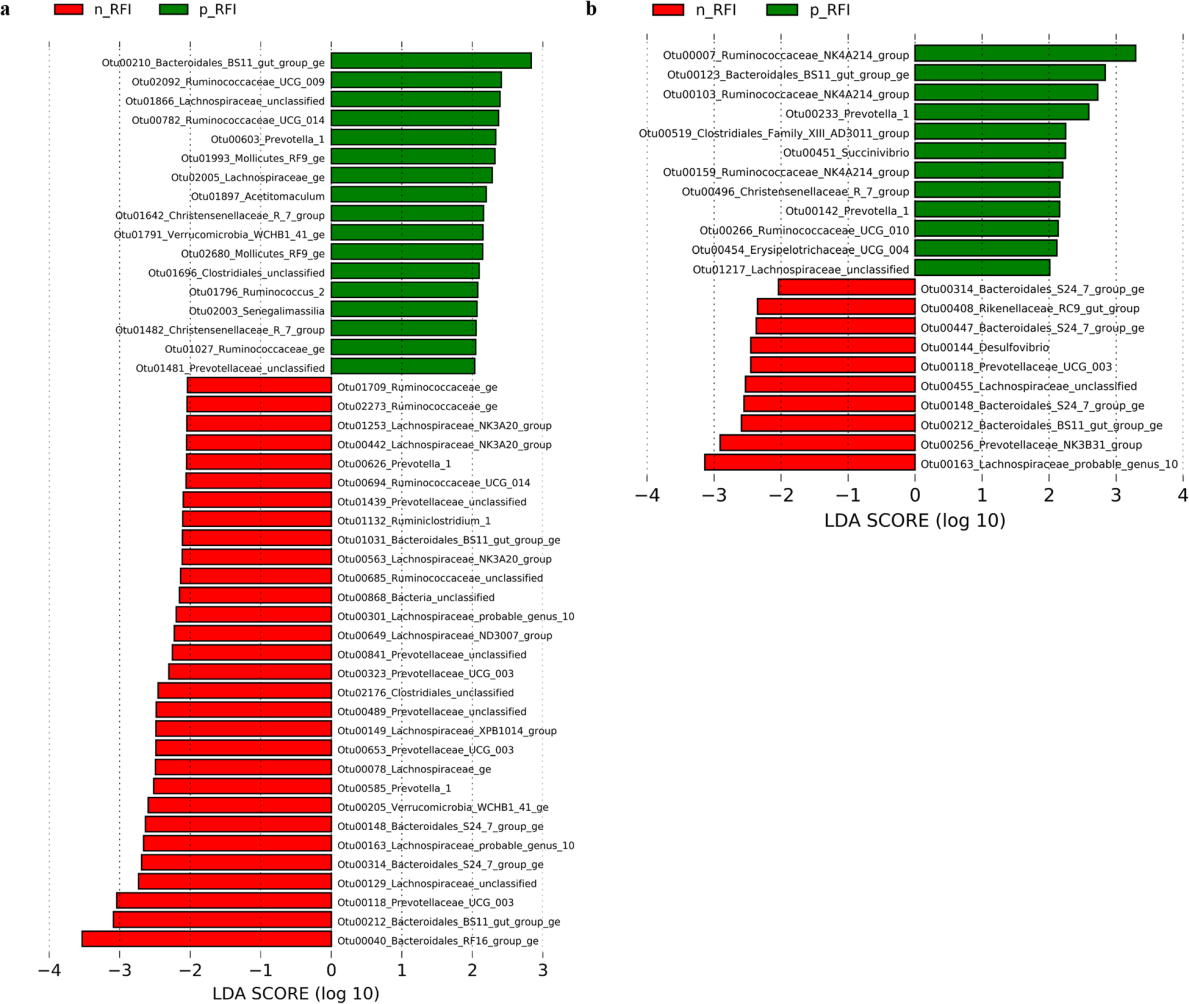
Biomarker bacterial OTUs identified by Linear discriminant analysis Effect Size (LEfSe, *P* < 0.05) in the liquid (**a**) and solid (**b**) fractions of rumen contents from Nellore steers showing low (p-RFI) or high (n-RFI) feed efficiency. Red bars represent enriched or exclusive OTUs in animals with high feed efficiency (n-RFI), while green bars represent enriched or exclusive OTUs in animals with low feed efficiency (p-RFI)

A Linear discriminant analysis of Effect Size (LEfSe) showed that 47 bacterial OTUs in the RL fraction of steers were associated with FE, of which 17 were increased or exclusive in the p-RFI steers (corresponding 0.37% of relative abundance to p-RFI and 0.042% to n-RFI), while 30 were increased or exclusive in the n-RFI steers (corresponding 1.32% of relative abundance to p-RFI and 3.54% to n-RFI) (Fig. 4a and Table 2). Twenty-two biomarker OTUs were identified in the RS fraction, with 12 being enriched or unique of p-RFI steers (corresponding 2.43% of relative abundance to p-RFI and 1.65% to n-RFI), whereas 10 were enriched or unique of n-RFI steers (corresponding 0.43% of relative abundance to p-RFI and 1.24% to n-RFI) (Fig. 4b and Table 2). Five bacterial OTU biomarkers (Otu00118, Otu00148, Otu00163, Otu00212, Otu00314) were common to both ruminal fractions (RL and RS) and all were increased in the most efficient animals (n-RFI) (Fig. 4 and Table 2). Most of these biomarkers belonged to the order Bacteroidales, including the family Prevotellaceae and genus *Prevotella*, as well as members of the order Clostridiales order, including the families Lachnospiraceae, Ruminococcaceae and Christensenellaceae (Fig. 4 and Table 2).

**Table 2:** Relative abundance of biomarker OTUs identified by LEfSe in the rumen of steers according to RFI

		Taxonomy^**a**^	p-RFI	n-RFI	***P-***value
Bacteria					
Rumen liquid	Otu00210	Bacteroidales BS11 gut group ge	0.1680	0.0101	0.0332
	Otu00603	*Prevotella* 1	0.0614	0.0207	0.0433
	Otu00782	Ruminococcaceae UCG 14	0.0428	0.0008	0.0097
	Otu01027	Ruminococcaceae ge	0.0201	0.0042	0.0073
	Otu01481	Prevotellaceae unclassified	0.0087	0.0025	0.0366
	Otu01482	Christensenellaceae R 7 group	0.0107	0.0017	0.0063
	Otu01642	Christensenellaceae R 7 group	0.0047	–	0.0078
	Otu01696	Clostridiales unclassified	0.0080	0.0008	0.0296
	Otu01791	Verrucomicrobia WCHB1 41 ge	0.0113	–	0.0078
	Otu01796	*Ruminococcus* 2	0.0053	0.0008	0.0461
	Otu01866	Lachnospiraceae unclassified	0.0067	–	0.0037
	Otu01897	*Acetitomaculum*	0.0033	–	0.0308
	Otu01993	Mollicutes RF9 ge	0.0033	–	0.0308
	Otu02003	*Senegalimassilia*	0.0040	–	0.0308
	Otu02005	Lachnospiraceae ge	0.0034	–	0.0308
	Otu02092	Ruminococcaceae UCG 9	0.0047	0.0008	0.0344
	Otu02680	Mollicutes RF9 ge	0.0033	–	0.0308
	Otu00040	Bacteroidales RF16 group ge	0.4565	1.0930	0.0047
	Otu00078	Lachnospiraceae ge	0.1190	0.1784	0.0404
	Otu00118	Prevotellaceae UCG 3	0.1459	0.3424	0.0192
	Otu00129	Lachnospiraceae unclassified	0.0653	0.1537	0.0453
	Otu00148	Bacteroidales S24 7 group ge	0.1176	0.1994	0.0279
	Otu00149	Lachnospiraceae XPB1014 group	0.0468	0.1130	0.0157
	Otu00163	Lachnospiraceae probable genus 10	0.0121	0.1013	0.0454
	Otu00205	Verrucomicrobia WCHB1 41 ge	0.1008	0.1479	0.0273
	Otu00212	Bacteroidales BS11 gut group ge	0.0457	0.2683	0.0335
	Otu00301	Lachnospiraceae probable genus 10	0.0100	0.0429	0.0488
	Otu00314	Bacteroidales S24 7 group ge	0.0532	0.1390	0.0129
	Otu00323	Prevotellaceae UCG 3	0.0381	0.0765	0.0279
	Otu00442	Lachnospiraceae NK3A20 group	0.0207	0.0344	0.0218
	Otu00489	Prevotellaceae unclassified	–	0.0652	0.0069
	Otu00563	Lachnospiraceae NK3A20 group	0.0080	0.0287	0.0467
	Otu00585	*Prevotella 1*	–	0.0833	0.0443
	Otu00626	*Prevotella 1*	0.0207	0.0494	0.0298
	Otu00649	Lachnospiraceae ND3007 group	0.0160	0.0503	0.0487
	Otu00653	Prevotellaceae UCG 3	0.0027	0.0760	0.0012
	Otu00685	Ruminococcaceae unclassified	0.0060	0.0151	0.0012
	Otu00694	Ruminococcaceae UCG 14	0.0120	0.0319	0.0126
	Otu00841	Prevotellaceae unclassified	0.0040	0.0337	0.0058
	Otu00868	Bacteria unclassified	0.0074	0.0343	0.0492
	Otu01031	Bacteroidales BS11 gut group ge	0.0020	0.0326	0.0320
	Otu01132	Ruminiclostridium 1	0.0040	0.0134	0.0050
	Otu01253	Lachnospiraceae NK3A20 group	0.0040	0.0117	0.0291
	Otu01439	Prevotellaceae unclassified	–	0.0210	0.0069
	Otu01709	Ruminococcaceae ge	0.0020	0.0084	0.0180
	Otu02176	Clostridiales unclassified	0.0007	0.0042	0.0320
	Otu02273	Ruminococcaceae ge	0.0007	0.0050	0.0161
Rumen solid	Otu00007	Ruminococcaceae NK4A214 group	1.4051	1.0663	0.0318
	Otu00103	Ruminococcaceae NK4A214 group	0.2949	0.1909	0.0218
	Otu00123	Bacteroidales BS11 gut group ge	0.2003	0.1735	0.0294
	Otu00142	*Prevotella* 1	0.0525	0.0216	0.0246
	Otu00159	Ruminococcaceae NK4A214 group	0.1357	0.1060	0.0281
	Otu00233	*Prevotella* 1	0.1041	0.0192	0.0129
	Otu00266	Ruminococcaceae UCG 10	0.0564	0.0275	0.0244
	Otu00451	*Succinivibrio*	0.0343	–	0.0308
	Otu00454	Erysipelotrichaceae UCG 4	0.0348	0.0034	0.0311
	Otu00496	Christensenellaceae R 7 group	0.0517	0.0266	0.0191
	Otu00519	Clostridiales Family XIII AD3011 group	0.0491	0.0174	0.0303
	Otu01217	Lachnospiraceae unclassified	0.0175	0.0008	0.0217
	Otu00118	Prevotellaceae UCG 3	0.0815	0.1299	0.0318
	Otu00144	*Desulfovibrio*	0.1101	0.1608	0.0359
	Otu00148	Bacteroidales S24 7 group ge	0.0724	0.1467	0.0145
	Otu00163	Lachnospiraceae probable genus 10	0.0594	0.2891	0.0457
	Otu00212	Bacteroidales BS11 gut group ge	0.0391	0.1197	0.0335
	Otu00256	Prevotellaceae NK3B31 group	0.0154	0.1712	0.0426
	Otu00314	Bacteroidales S24 7 group ge	0.0087	0.0267	0.0159
	Otu00408	Rikenellaceae RC9 gut group	0.0073	0.0459	0.0403
	Otu00447	Bacteroidales S24 7 group ge	0.0129	0.0596	0.0366
	Otu00455	Lachnospiraceae unclassified	0.0195	0.0884	0.0401
Archaea					
Rumen liquid	Otu0012	*Methanobrevibacter*	2.3912	0.0787	0.0220
	Otu0016	*Methanobrevibacter*	0.3747	0.1574	0.0343
	Otu0044	*Methanobrevibacter*	0.0493	–	0.0249
Fungi					
Rumen liquid	Otu000015	Neocallimastigaceae unclassified	1.2594	0.1456	0.0082
	Otu000057	Neocallimastix unclassified	0.2374	0.0987	0.0353
	Otu000004	*Piromyces* sp	10.3505	18.7319	0.0281
	Otu000052	Fungi unclassified	0.1377	1.4443	0.0311
	Otu000097	Neocallimastigaceae unclassified	0.0173	0.1382	0.0399
	Otu000138	Fungi unclassified	–	0.0826	0.0443
	Otu000167	Fungi unclassified	–	0.2110	0.0443
	Otu000197	*Piromyces* sp	–	0.0760	0.0179
	Otu000233	Fungi unclassified	–	0.0465	0.0443
	Otu000360	*Caecomyces communis*	–	0.0264	0.0443
	Otu000442	Fungi unclassified	–	0.0601	0.0179
	Otu000447	*Piromyces* sp	–	0.0556	0.0443
	Otu000553	Fungi unclassified	–	0.0326	0.0443
	Otu000827	Fungi unclassified	–	0.0388	0.0443
Rumen solid	Otu000004	*Piromyces* sp	4.4228	11.5187	0.0047
	Otu000005	*Piromyces* sp	3.6348	10.0991	0.0102
	Otu000068	Neocallimastigaceae unclassified	–	0.0540	0.0354
	Otu000197	*Piromyces* sp	–	0.0889	0.0132
	Otu000232	*Caecomyces communis*	0.0046	0.0944	0.0185
	Otu000243	*Orpinomyces* sp	–	0.0525	0.0354
	Otu000336	*Piromyces* sp	0.0084	0.0532	0.0223
	Otu000370	*Piromyces* sp	0.0042	0.0414	0.0185
	Otu000404	*Piromyces* sp	0.0044	0.0301	0.0456
	Otu000510	*Caecomyces communis*	–	0.0418	0.0354
	Otu000559	Neocallimastigaceae unclassified	–	0.0179	0.0354
	Otu000666	Neocallimastigaceae unclassified	–	0.0177	0.0354

Bolded biomarker OTUs indicates as increase in Nellore steers showing high (n-RFI) feed efficiency

^a^Taxonomy for each OTU is given at the highest classifiable level

### Richness and specific OTUs of the Archaeal community are associated with the p-RFI and n-RFI phenotypes

The alpha diversity analysis of RL and RS archaeal communities demonstrated that Simpson’s and Shannon’s diversity did not vary significantly in response to RFI group (t-test *P* > 0.05), although the Chao1 richness of both rumen fractions from n-RFI steers (45.18 ± 11.1) was significantly greater than that found in p-RFI steers (36.22 ± 8.12) (Table 1). Beta diversity analysis, as summarized in NMDS plots of Bray-Curtis dissimilarities, showed differences between the RL and RS communities with both groups clustering separately (ANOSIM, *P* < 0.001). However, no differences were observed in the RL and RS archaeal communities of p-RFI and n-RFI steers (Fig. 1b).

Taxonomic composition analyses of the archaeal community revealed 78 unique OTUs in the rumen contents (liquid and solid phase) of Nellore steers. All OTUs were assigned to the phylum Euryarchaeota, with 82.26 ± 0.47% of these classifying to the family Methanobacteriacea and 15.63 ± 0.53% assigned to the family Thermoplasmatales Incertae Sedis. The less abundant groups of ruminal archaea accounted for 1.89 ± 0.01% of the sequences and were classified to the family Methanosarcinaceae (Supplementary Fig. S3).

**Fig. 5:**
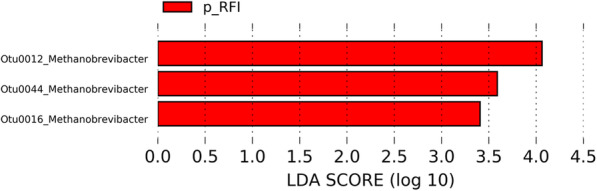
Biomarker archaeal OTUs identified by Linear discriminant analysis Effect Size (LEfSe, *P* < 0.05) in the liquid rumen fraction of Nellore steers showing low (p-RFI) or high (n-RFI) feed efficiency. Red bars represent enriched or exclusive OTUs in animals with low feed efficiency (p-RFI)

We found that 17.43 ± 5.82% and 16.27 ± 4.41% of the OTUs in the RL and RS, respectively, could not be assigned at the genus level (Supplementary Fig. S4). The archaeal community in the ruminal contents were dominated by members of the genus *Methanobrevibacter* (77.23 ± 6% in RS), followed by *Methanosphaera* (3.21 ± 1.67%) and *Methanimicrococcus* (1.65 ± 1.27%) (Supplementary Fig. S4). When considering FE groups, the relative abundance of *Methanimicrococcus* was, on average, two-fold higher in n-RFI steers than in p-RFI steers (White’s non-parametric t-test, *P* < 0.05) (Fig. 2). Archaeal biomarker OTUs in each FE group were identified only in the rumen liquid fraction by the LEfSe analysis. The three biomarker OTUs were classified as belonging to the genus *Methanobrevibacter* and were either increased or exclusive of p-RFI steers (2.82% of relative abundance to p-RFI and 0.24% to n-RFI) (Fig. 5 and Table 2).

### Specific high abundance fungal OTU are associated with the p-RFI and n-RFI phenotypes

The fungal intra-community diversity (alpha diversity) did not vary between RFI groups (*t*-test, *P* > 0.05) (Table 1). Beta diversity analysis (Bray-Curtis dissimilarity index) represented on an NMDS plot showed that the fungal communities from the two efficiency groups (p-RFI and n-RFI) and ruminal fractions (RL and RS) were not significantly different (ANOSIM, *P* > 0.001) (Fig. 1c).

Taxonomic composition analyses of the fungal community revealed 1,795 unique OTUs in the rumen contents (RL and RS). All OTUs classified were assigned to the family Neocallimastigaceae and nearly 25.46 ± 10.35% and 16.21 ± 4.55% of the OTUs in the RL and RS, respectively, could not be assigned to the genus level. In decreasing order of relative abundance, the assigned genera were *Caecomyces*, *Piromyces, Orpinomyces, Cyllamyces, Neocallimastix, Anaeromyces,* and *Buwchfawromyces* (Supplementary Table S3), with the *Buwchfawromyces* genus being almost five-fold higher in the RS fraction of n-RFI steers than in the p-RFI steers (White’s non-parametric *t*-test, *P* < 0.05) (Fig. 2).

**Fig. 6:**
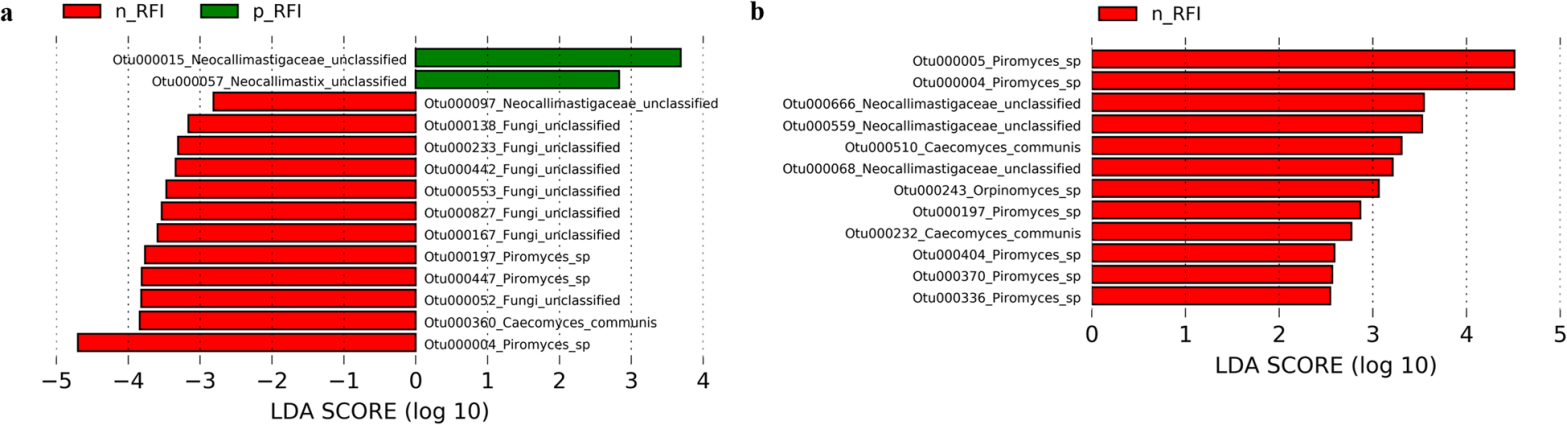
Biomarker fungal OTUs identified by Linear discriminant analysis Effect Size (LEfSe, *P* < 0.05) in the liquid (**a**) and solid (**b**) fractions of rumen contents from Nellore steers showing low (p-RFI) or high (n-RFI) feed efficiency. Red bars represent enriched or exclusive OTUs in animals with higher feed efficiency (n-RFI), while green bars represent enriched or exclusive OTUs in animals with lower feed efficiency (p-RFI)

Our LEfSe showed that 14 fungal OTUs in the RL fraction of steers were associated with FE phenotype, of which 2 were increased in the p-RFI steers (corresponding to 1.50% of the relative abundance in p-RFI steers and 0.24% in n-RFI steers), while 12 were increased or exclusive to n-RFI steers (corresponding to 10.50% of the relative abundance in p-RFI steers and 20.94% in n-RFI steers) (Fig. 6a and Table 2). In the RS phase, 12 biomarker OTUs were identified, all enriched or unique to n-RFI steers (corresponding 8.08% of relative abundance to p-RFI and 22.11% to n-RFI) (Fig. 6b and Table 2). Two biomarker fungal OTUs (*Piromyces* genus) were common to both ruminal fractions (RL and RS): Otu000004, which was among the most abundant OUT in the fungal community and was enriched in the n-RFI steers, and Otu000197, which was only detected in n-RFI steers (Fig. 6 and Table 2). Among the 24 fungal biomarkers identified, 7 belonged to the genus *Piromyces*, 5 to unclassified Neocallimastigaceae, 3 to *Caecomyces communis*, one to the *Orpinomyces*, and one to the *Neocallimastix*; 7 OTUs could not be classified at any taxonomical level (Fungi unclassified) (Fig. 6 and Table 2).

### Functional prediction and correlations between the bacterial community and ruminal fermentation products

To gain a better understanding of the functional role of the rumen bacterial community, as it relates to FE groups, we performed a prediction of metabolic functions for both solid and liquid phases and found 244 functional categories between the FE groups (Supplementary Table S4). No significant differences were detected for the predicted metabolic functions of Nellore steers with distinct FE phenotypes. These results are in agreement with the lack of differences in the quantification of rumen fermentation products between FE groups observed in this study. We also found that the molar proportion of acetic, propionic, succinic, butyric, isobutyric, valeric, and isovaleric acids, acetate-to-propionate ratio, total concentration of short-chain volatile fatty acids (VFA), ammonia concentration, and ruminal pH did not vary between the p-RFI and n-RFI steers (t-test, *P* > 0.05) (Supplementary Table S5).

**Fig. 7:**
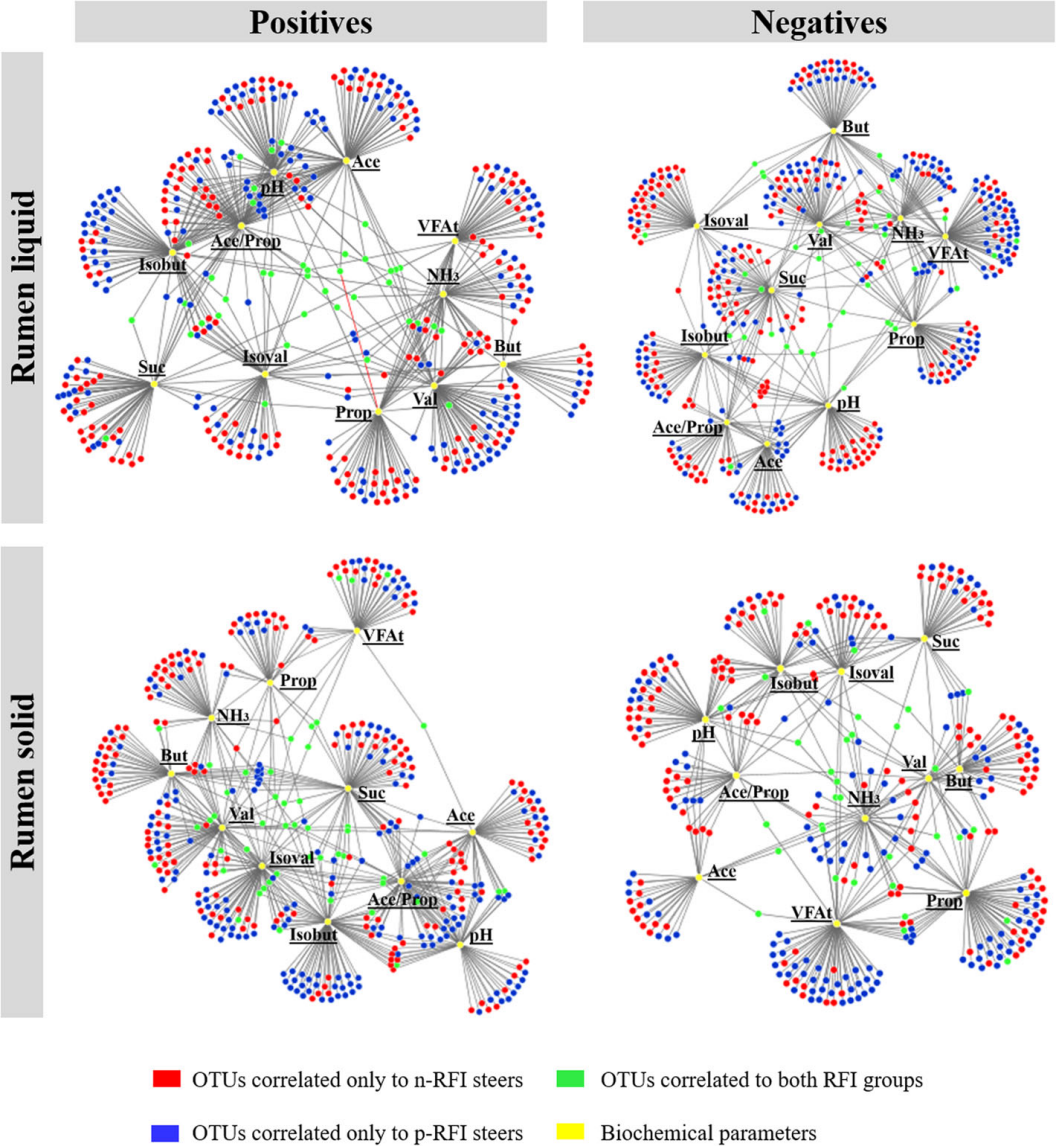
Correlation network between liquid and solid bacterial OTUs and ruminal fermentation variables according to each RFI group. The edges represent significant associations, as determined by Spearman’s rank correlation (*P* < 0.05). These analyses were performed using OTUs that were shared by at least 50% of the steers in each feed efficiency group. Biochemical parameters: molar proportion of acetic, Ace; propionic, Prop; succinic, Suc; butyric, But; isobutyric, Isobut; valeric, Val; and isovaleric acids, Isoval; acetate-to-propionate ratio, Ace/Prop; total concentration of these volatile fatty acids, VFAt; ammonia concentration, NH_3_; and ruminal pH)

However, our microbial-metabolite network analysis revealed significant associations (positive and negative) between the 638 bacterial OTUs in the RL fraction and the 544 OTUs in the RS fraction with ruminal fermentation products (e.g., VFAs and ruminal NH_3_), which were represented by correlations (nodes connected by an edge) with specific fermentation products (Fig. 7). In the RL phase, 193 p-RFI steer OTUs, 183 n-RFI steer OTUs, and 44 OTUs shared by both groups were positively associated with at least one ruminal fermentation product. The p-RFI steers had nearly two times more OTUs correlated to the concentration of acetic acid, isobutyric acid, and ruminal pH than the n-RFI steers. However, the opposite was observed for ammonia concentration and total VFA in the n-RFI steers, where the number of OTUs correlating with these parameters was higher compared to the OTUs in the RL phase of the p-RFI steers (Fig. 7). In the RS phase 183 and 180 bacterial OTUs from the p-RFI and n-RFI steers, respectively, and 57 OTUs shared by both groups were positively associated with at least one ruminal variable. Isobutyric acid and ruminal pH were more correlated with OTUs from the p-RFI steers than the n-RFI steers, while ammonia concentration was more correlated with OTUs from n-RFI steers (Fig. 7).

Analysis of negative correlations between microbial taxa and ruminal fermentation products (e.g., VFAs, NH_3_) revealed that 198 OTUs from p-RFI steers and 200 OTUs from n-RFI steers, as well as 39 OTUs shared by both groups, were negatively associated with at least one biochemical parameter. The concentration of propionic acid and total VFAs was more negatively correlated with OTUs in p-RFI steers, while a greater number of negative associations were observed for isovaleric acid and ruminal pH and taxa identified in n-RFI steers (Fig. 7). In the solid fraction, there were 148 OTUs in p-RFI steers and 163 OTUs in n-RFI steers, in addition to 35 OTUs from both FE groups, associated with at least one ruminal fermentation parameter. Total VFA was negatively correlated with more OTUs in the p-RFI steers than in n-RFI steers, while ruminal pH was more negatively correlated with OTUs linked to the n-RFI steers (Fig. 7).

Among the most abundant OTUs showing positive or negative correlations with fermentation parameters, we observed a predominance of OTUs from genera in the families Prevotellaceae, Ruminococcaceae, Rikenellaceae, Lachnospiraceae*,* and Christensenellaceae in the bacterial communities of both RL and RS phases (Supplementary Tables S6-S9). Taxonomic differences were also observed between the most abundant OTUs correlated with steers in the p-RFI or n-RFI groups. The most abundant OTUs that were positively correlated with total VFAs were classified to the genus *Papillibacter*, *Treponema*, and the families Bacteroidales, Ruminococcaceae, Prevotellaceae UCG-004, and unclassified Lachnospiraceae in the p-RFI steers, while in the n-RFI steers the most abundant OTUs were assigned to the order Bacteroidales BS1 and S24–7, the families Rikenellaceae RC9, Anaerolineaceae, Christensenellaceae R-7, Lachnospiraceae XPB1014, and the genera *Prevotella*, *Phocaeicola*, and *Anaerotruncus* (Supplementary Table S6).

## Discussion

Here we investigated the composition of the ruminal microbial communities of high and low FE Nellore beef cattle raised under tropical conditions to identify if specific ruminal microbiota are correlated to FE. Although the steers in this study were classified into different FE groups based on their residual feed intake (RFI), a measure that has the advantage of being independent of animal growth and weight [43, 44], we note that RFI has some limitations since it depends on the confinement of the herd for long periods (≥ 90 d), requires daily recording of feed consumption and feed refusals, and weight assessments of the animals. In addition, if animals are housed in groups, individual feed intake can only be verified using specialized equipment (eg: GrowSafe). In tropical countries such as Brazil, limitations are accentuated by beef cattle that are raised predominantly on pasture and very few farmers have access to the infrastructure needed to measure RFI on a commercial scale.

Our study demonstrates that Nellore steers with a lower FE harbor a ruminal microbiota with an increased Firmicutes/Bacteroidetes (F/B) ratio. Changes in the overall composition of the gut microbiota reflecting an increased F/B ratio have been implicated in certain animal phenotypes, including higher fat deposition in the blood and tissues of humans and mice [45] and increased milk fat in dairy cows [10]. Nonetheless, differences in the F/B ratio can not be generalized across animal studies as relative abundances might be influenced by various factors, such as unbalanced sampling, sample processing methods, inter-individual variation, diet composition and feed additives, resulting in conflicting results [14, 46, 47].

We also identified a higher relative abundance (0.13%) of bacteria within the family Peptostreptococcaceae (phylum Firmicutes) in the rumen of low efficient animals (p-RFI steers). Despite their low abundance in the rumen, members of this group have been associated with low nitrogen retention in Nellore steers [48]. This family includes members of the hyper ammonia-producing bacteria (HAB) [49], which have exceptionally high deamination activity and are thought to contribute to ammonia overflow and inneficient nitrogen retention in ruminants [50]. Although no differences in ruminal ammonia nitrogen were observed between the p-RFI and n-RFI steers in the current study, urinary N excretion and N retention deserve further investigation in future studies.

Our results also revealed specific OTUs belonging to the families Lachnospiraceae and Ruminococcaceae and the genus *Prevotella* that are associated with both n-RFI (more efficient) and p-RFI (less efficient) steers (Table 2 and Supplementary Table S3). These taxa are among the most dominant bacteria in the rumen microbiome and show remarkable functional and genetic diversity, occupying various ecological niches in the rumen ecosystem [51, 52]. For example, the genus *Prevotella* is one of the most abundant taxa in the rumen of cattle and contains numerous metabolically versatile bacterial species that can grow on starch, protein, peptides, hemicellulose, and pectin [27]. Our data support previous observations indicating that members of these microbial groups can be both positively and negatively correlated with FE in beef and dairy cattle [2, 12, 16, 53]. Importantly, bacteria in the family Lachnospiraceae appear to have greater relative abundance in Nellore steers with low nitrogen retention and in beef cattle showing p-RFI phenotype [48, 54], and a significant negative correlation was also reported for the Ruminococcaceae in the microbiomes of inefficient Holstein cows [2].

These findings indicate that some microbial taxa in the n-RFI animals may contribute more efficiently to the metabolism and energy utilization of dietary components. For example, *Megasphaera elsdenii* and *Coprococcus catus* were associated with greater production of butyrate and propionate in the rumen microbiome of efficient Holstein cows [2] and functional prediction of bacterial OTUs related to feed efficiency in beef steers indicated a higher abundance of transporters for the uptake of nutrients [19]. In addition, studies that evaluated the rumen microbiota of *Bos taurus* reported that five *Prevotella* OTUs were more abundant in p-RFI cows and seven OTUs more abundant in n-RFI cows, suggesting that the effects on the host for these organisms are species/OTU-specific [12]. Moreover, Chiquette et al. [55] demonstrated that inoculation of *Prevotella* into the rumen of dairy cows leads to decreased lactate production and increased milk fat yield. In contrast, Jami et al. [10] found a strong and significant negative correlation between the abundance of *Prevotella* and milk fat production in Israeli Holstein Friesian lactating cows. More recently, Brooke et al. [56] reported that *Prevotella copri* was the most highly enriched OTU in the fecal microbiota of high FE beef steers, accounting for 3.0% of the relative abundance in high-efficiency animals and only 0.14% relative abundance in low-efficiency steers. These results suggest that specific taxa might be positively correlated with rumen fermentation variables in high-efficiency steers and could serve as potential biomarkers for FE in Nellore cattle, similar to the findings reported for *Bos taurus* cows.

Although no differences were observed in the predicted functional profile of the rumen bacterial community, our analyses show that different bacterial OTUs are correlated with individual fermentation products for each FE group, and most of these OTUs belong to the same taxonomic groups. Previously, Guan and coworkers reported significantly higher concentrations of butyrate and valerate in the rumen of n-RFI steers, but these changes in VFA concentrations were not linked to specific rumen microbes [13]. Taken together, these findings reinforce the idea that FE is a complex trait with specific ruminal bacteria potentially having different contributions to rumen metabolism and host physiology linked to the efficiency of feed utilization. Recent studies using culture-dependent and culture-independent (e.g. RNA-seq) methods have sought to improve the characterization of functional groups involved in ruminal fermentation, thus expanding our understanding of their role in this ecosystem [50, 57, 58].

In addition to the complex bacterial community, which is responsible for hydrolyzing non-structural and structural carbohydrates, proteins, peptides, amino amides, and lipids into VFAs, ammonia, hydrogen, and CO_2_, the archaeal community also plays important a role in the energy retention balance in ruminants, serving as an electron sink that drives the direction of ruminal fermentation [59]. Our results indicate that p-RFI Nellore steers exhibit a lower archaeal richness in their rumen, suggesting a more specialized archaeal community with different affinities for H_2_ compared to the n-RFI steers. However, it is important to note that methane emissions were not measured in this study, and the alpha diversity indices (e.g., Shannon, Simpson) did not differ between methanogenic communities in n-RFI and p-RFI steers. This is in contrast to published studies that report different outcomes for the compositional variation of methanogenic communities and the abundance of archaea in low and high FE cattle [9, 14, 15, 59–61]. The discrepancies among studies is likely because methane production is a complex process involving various members of the rumen microbial community (e.g., bacteria, protozoa, fungi) that generate the main substrates for methanogenesis, such as H_2_ and CO_2_ [60].

In agreement with results reported for *Bos taurus* [14, 18], our findings reveal that *Methanobrevibacter* was the most abundant genus of archaea in the rumen of Nellore steers, followed by *Methanosphaera* and *Methanimicrococcus*. It is also significant that the genus *Methanobrevibacter* was increased in p-RFI steers. These methanogenic archaea use the hydrogenotrophic pathway for methane production and utilize H_2_ or formic acid as electron donors [62]. Although some ruminal archaea (e.g. members of the order *Methanosarcinales* and the genus *Methanosphaera*) that use the methylotrophic or the acetoclastic pathways were also found in our study, we are unable to speculate about the relationship between different methane production pathways and FE in the Nellore steers.

The anaerobic fungi are long thought to play an important role in fiber degradation in the rumen and are recognized as one of the first colonizers of lignocellulosic substrates in ruminant diets [63]. Because the fungal mycelia can penetrate and physically disrupt feed particles, more surface area is exposed for microbial colonization, thereby improving degradation of the plant biomass [64]. Our analysis of the fungal community shows no differences in beta diversity between the solid and liquid fractions, indicating that the fungal populations attached to the feed particles are similar to those in the planktonic state. To our knowledge, this is the first work describing the structure and diversity of the anaerobic fungi community in the rumen solid and liquid fractions of Nellore cattle.

Our fungal taxonomic analysis identified the genus *Buwchfawromyces* as being more highly distributed in the RS fraction of the more FE steers. *Buwchfawromyces* is a new genus of anaerobic fungi belonging to the order *Neocallimastigales* (phylum *Neocallimastigomycota*) that was isolated from fecal samples of buffalo, sheep, cattle, and horses [65]. This fungus produces a monocentric thallus, spherical to ovoid sporangia, and shows an extensive rhizoidal system. However, little information is available about its physiology and further studies are warranted to investigate the metabolic potential of the isolated strains.

A more detailed analysis of the fungal OTUs revealed that the Otu000004, classified to the *Piromyces* genus, was present in high proportions (varying on average from 4.42% to 18.73%) in the RL and RS fractions in both FE groups, and was significantly more abundant in the high efficient steers (n-RFI), relative to low efficient steers (p-RFI). Wang et al. [66] observed that inoculation of *Piromyces* sp. CN6 CGMCC 14449, isolated from the rumen of Xinong Saanen dairy goats, increased the in vitro digestibility of dry matter, neutral detergent fiber, and acid detergent fiber in maize silage. This emphasizes the need for continued efforts aiming at isolating and characterizing rumen anaerobic fungi that could be associated with FE in beef cattle, including in Nellore steers. These fungal populations could help improve the utilization of insoluble substrates, especially in ruminants fed forage-rich diets [63], such as the Nellore cattle that are raised mostly on grasses in the tropics.

## Conclusions

The findings reported here provide insights into the specific differences in the bacterial, archaeal, and fungal ruminal communities of Nellore steers with high and low FE phenotypes. Individuals with a larger relative abundance of Bacteroidetes and lower relative abundance of Firmicutes and metanogenic archaea were more efficient at feed utilization. We have identified some candidate biomarkers representing important functional groups involved in the ruminal fermentation of dietary compounds. However, due to the intrinsic inter- and intra-individual variation in microbiota and the complexity of animal traits such as feed efficiency, follow-up validation using larger cohorts of cattle will be needed to corroborate these findings. Future studies, including analysis of the genetic and functional capabilities of rumen microorganisms using approaches such as metagenomics, metatranscriptomics, metaproteomics, and metabolomics can provide a greater understanding of the relationship between the ruminal microbiota and FE. In addition, efforts targeting the isolation, characterization, and quantification of ruminal strains that vary in abundance and diversity between n-RFI and p-RFI Nellore steers will be of value to understand the metabolic attributes and ecological functions of microorganisms with potential causal roles in the FE of beef cattle.

## Supplementary Information

**Additional file 1: Supplementary Table S1.** Residual feed intake (RFI) of the 129 Nellore steers evaluated in this study. **Supplementary Table S2.** Summary of sequencing data (Bacteria, Archaea and Fungi) derived from ruminal contents of Nellore steers according to ruminal phase and RFI group. **Supplementary Fig. S1.** Ruminal bacterial composition at the phyla level of rumen liquids and rumen solids samples. **Supplementary Fig. S2.** Ruminal bacterial composition at the family level of rumen liquids and rumen solids samples. **Supplementary Fig. S3.** Composition of the ruminal archaea at the class, order and family levels. **Supplementary Fig. S4.** Ruminal archaeal composition at the genus level of rumen liquids and rumen solids samples. **Supplementary Table S3.** Ruminal fungal composition at genus-level according to RFI group. **Supplementary Table S4.** Relative abundance (%) of the functional categories predicted for the bacterial microbiota in liquid and solid ruminal fractions of Nellore steers showing high (n-RFI) and low (p-RFI) feed efficiency. **Supplementary Table S5.** Fermentation profile of the ruminal liquid of Nellore steers according to RFI group. **Supplementary Table S6.** Relative abundance of the most abundant bacterial OTUs showing positive correlation with ruminal fermentation parameters in p-RFI steers. **Supplementary Table S7.** Relative abundance of the most abundant bacterial OTUs showing positive correlation with ruminal fermentation parameters in n-RFI steers. **Supplementary Table S8.** Relative abundance of the most abundant bacterial OTUs showing negative correlation with ruminal fermentation parameters in p-RFI steers. **Supplementary Table S9.** Relative abundance of the most abundant bacterial OTUs showing negative correlation with ruminal fermentation parameters in n-RFI steers.

## Data Availability

All DNA sequences have been deposited in the NCBI’s Sequence Read Archive (SRA - https://www.ncbi.nlm.nih.gov/sra) under BioProject accession number PRJNA512996.

## Abbreviations

ADG: Average daily gain
BW: Body weight
FE: Feed efficiency
CEUAP/UFV: Universidade Federal de Viçosa Ethics Committee on Production Animal Use
CEUA-IZ: Instituto de Zootecnia Ethics Committee on Animal Use
DMI: Dry matter intake
F/B: Firmicutes to bacteroidetes
ITS1: Internal transcribed spacer 1
LDA: Logarithmic Linear Discrimination Analysis
LEfSe: Linear discriminant analysis effect size
NMDS: Non-metric multidimensional scaling
n-RFI: Negative-RFI
OTU: Operational taxonomic unit
PICRUSt: Phylogenetic Investigation of Communities by Reconstruction of Unobserved States
p-RFI: Positive-RFI
RFI: Residual feed intake
RL: Rumen liquid
RS: Rumen solids
SD: Standard deviation
SRA: Sequence Read Archive
STAMP: Statistical Analysis of Taxonomic and Functional Profiles
VFA: Volatile fatty acids
