# Supplementary material for: Assessing the relationship between the rumen microbiota and feed efficiency in Nellore steers
Source: J Anim Sci Biotechnol. 2021 Jul 15;12:79. doi: 10.1186/s40104-021-00599-7 (PMC8281616; doi:10.1186/s40104-021-00599-7)
Supplement: Supplementary file 1 — Additional file 1: Supplementary Table S1. Residual feed intake (RFI) of the 129 Nellore steers evaluated in this study. Supplementary Table S2. Summary of sequencing data (Bacteria, Archaea and Fungi) derived from ruminal contents of Nellore steers according to ruminal phase and RFI group. Supplementary Fig. S1. Ruminal bacterial composition at the phyla level of rumen liquids and rumen solids samples. Supplementary Fig. S2. Ruminal bacterial composition at the family level of rumen liquids and rumen solids samples. Supplementary Fig. S3. Composition of the ruminal archaea at the class, order and family levels. Supplementary Fig. S4. Ruminal archaeal composition at the genus level of rumen liquids and rumen solids samples. Supplementary Table S3. Ruminal fungal composition at genus-level according to RFI group. Supplementary Table S4. Relative abundance (%) of the functional categories predicted for the bacterial microbiota in liquid and solid ruminal fractions of Nellore steers showing high (n-RFI) and low (p-RFI) feed efficiency. Supplementary Table S5. Fermentation profile of the ruminal liquid of Nellore steers according to RFI group. Supplementary Table S6. Relative abundance of the most abundant bacterial OTUs showing positive correlation with ruminal fermentation parameters in p-RFI steers. Supplementary Table S7. Relative abundance of the most abundant bacterial OTUs showing positive correlation with ruminal fermentation parameters in n-RFI steers. Supplementary Table S8. Relative abundance of the most abundant bacterial OTUs showing negative correlation with ruminal fermentation parameters in p-RFI steers. Supplementary Table S9. Relative abundance of the most abundant bacterial OTUs showing negative correlation with ruminal fermentation parameters in n-RFI steers. [file 40104_2021_599_MOESM1_ESM.docx]

**Supplementary Material**

**Supplementary Table S1** Residual feed intake (RFI) of the 129 Nellore steers evaluated in this study^a^

| **Animal number** | **RFI** | **Efficiency group^b^** |
| --- | --- | --- |
| 2143588 | -2.285 | 1 |
| **2143508** | **-1.773** | **1** |
| **2143564** | **-1.625** | **1** |
| 2143527 | -1.600 | 1 |
| **2143673** | **-1.583** | **1** |
| **2143674** | **-1.463** | **1** |
| 2143679 | -1.430 | 1 |
| 2143551 | -1.187 | 1 |
| **2143576** | **-1.185** | **1** |
| 2143616 | -0.947 | 1 |
| 2143573 | -0.923 | 1 |
| 2143683 | -0.912 | 1 |
| 2143615 | -0.887 | 1 |
| 2143609 | -0.880 | 1 |
| 2143634 | -0.871 | 1 |
| **2143657** | **-0.838** | **1** |
| 2143502 | -0.783 | 1 |
| 2143688 | -0.736 | 1 |
| 2143511 | -0.734 | 1 |
| **2143695** | **-0.718** | **1** |
| **2143552** | **-0.718** | **1** |
| **2143501** | **-0.703** | **1** |
| 2143681 | -0.695 | 1 |
| 2143607 | -0.686 | 1 |
| 2143610 | -0.653 | 1 |
| 2143542 | -0.617 | 1 |
| 2143691 | -0.540 | 2 |
| 2143549 | -0.490 | 2 |
| 2143623 | -0.472 | 2 |
| 2143589 | -0.461 | 2 |
| 2143506 | -0.456 | 2 |
| 2143624 | -0.441 | 2 |
| 2143665 | -0.421 | 2 |
| 2143606 | -0.396 | 2 |
| 2143539 | -0.391 | 2 |
| 2143633 | -0.388 | 2 |
| 2143554 | -0.357 | 2 |
| 2143505 | -0.345 | 2 |
| 2143612 | -0.345 | 2 |
| 2143583 | -0.324 | 2 |
| **2143672** | **-0.284** | **2** |
| 2143619 | -0.274 | 2 |
| 2143522 | -0.263 | 2 |
| 2143561 | -0.229 | 2 |
| 2143587 | -0.212 | 2 |
| 2143510 | -0.203 | 2 |
| 2143500 | -0.202 | 2 |
| 2143671 | -0.173 | 2 |
| **2143641** | **-0.131** | **2** |
| 2143670 | -0.107 | 2 |
| 2143536 | -0.101 | 2 |
| **2143565** | **-0.100** | **2** |
| 2143680 | -0.086 | 2 |
| 2143507 | -0.079 | 2 |
| 2143557 | -0.071 | 2 |
| 2143661 | -0.070 | 2 |
| 2143563 | -0.065 | 2 |
| 2143685 | -0.016 | 2 |
| 2143620 | -0.010 | 2 |
| 2143519 | -0.004 | 2 |
| 2143586 | 0.000 | 2 |
| 2143520 | 0.053 | 2 |
| 2143636 | 0.057 | 2 |
| 2143669 | 0.060 | 2 |
| 2143639 | 0.111 | 2 |
| 2143687 | 0.116 | 2 |
| 2143627 | 0.117 | 2 |
| 2143560 | 0.131 | 2 |
| 2143582 | 0.137 | 2 |
| **2143558** | **0.150** | **2** |
| 2143566 | 0.164 | 2 |
| 2143660 | 0.168 | 2 |
| 2143656 | 0.168 | 2 |
| 2143602 | 0.175 | 2 |
| 2143638 | 0.182 | 2 |
| 2143626 | 0.194 | 2 |
| 2143543 | 0.200 | 2 |
| 2143614 | 0.204 | 2 |
| **2143585** | **0.222** | **2** |
| 2143694 | 0.233 | 2 |
| 2143675 | 0.262 | 2 |
| 2143667 | 0.303 | 2 |
| **2143574** | **0.309** | **2** |
| 2143530 | 0.315 | 2 |
| 2143625 | 0.315 | 2 |
| 2143618 | 0.323 | 2 |
| 2143567 | 0.344 | 2 |
| 2143556 | 0.390 | 2 |
| 2143596 | 0.397 | 2 |
| 2143547 | 0.399 | 2 |
| 2143611 | 0.415 | 2 |
| 2143590 | 0.418 | 2 |
| 2143658 | 0.431 | 2 |
| 2143662 | 0.432 | 2 |
| 2143642 | 0.444 | 2 |
| **2143668** | **0.447** | **2** |
| 2143617 | 0.454 | 2 |
| **2143562** | **0.481** | **2** |
| 2143584 | 0.487 | 2 |
| **2143529** | **0.558** | **2** |
| 2143631 | 0.583 | 2 |
| 2143664 | 0.598 | 2 |
| 2143579 | 0.600 | 2 |
| 2143608 | 0.624 | 3 |
| 2143682 | 0.628 | 3 |
| 2143650 | 0.647 | 3 |
| 2143684 | 0.669 | 3 |
| **2143509** | **0.698** | **3** |
| 2143630 | 0.726 | 3 |
| 2143605 | 0.736 | 3 |
| **2143622** | **0.742** | **3** |
| 2143645 | 0.757 | 3 |
| **2143535** | **0.782** | **3** |
| 2143678 | 0.799 | 3 |
| 2143621 | 0.802 | 3 |
| 2143523 | 0.858 | 3 |
| 2143593 | 0.898 | 3 |
| 2143516 | 0.938 | 3 |
| 2143666 | 1.000 | 3 |
| **2143643** | **1.029** | **3** |
| **2143581** | **1.197** | **3** |
| **2143533** | **1.243** | **3** |
| 2143663 | 1.289 | 3 |
| **2143646** | **1.350** | **3** |
| 2143628 | 1.464 | 3 |
| 2143568 | 1.512 | 3 |
| **2143524** | **1.792** | **3** |
| 2143514 | 1.938 | 3 |
| **2143696** | **1.993** | **3** |

^a^Bold lines represent the 27 steers selected for evaluation in this study

^b^1: RFI < - 0.6 2: - 0.6 ≥ RFI ≤ 0.6 3: RFI > 0.6

**Supplementary Table S2** Summary of sequencing data (Bacteria, Archaea and Fungi) derived from ruminal contents of Nellore steers according to ruminal phase and RFI group^a^

|  |  |  |  |  | **After filtering and clean-up** | | **After normalization** | | **After cut off^b^** | |
| --- | --- | --- | --- | --- | --- | --- | --- | --- | --- | --- |
|  |  | **Steers (n)** | **Good's coverage** | **Good's coverage after normalization** | **Reads** | **OTUs** | **Reads** | **OTUs** | **Reads** | **OTUs** |
| **BACTERIA** |  |  |  |  |  |  |  |  |  |  |
| **Liquid** | **p-RFI** | 15 | 0.991 ± 0.003 | 0.954 ± 0.007 | 38,836 ± 14,279 | 1,441 ± 188 | 9,973 ± 72 | 1,068 ± 126 | 8,627 ± 417 | 706 ± 66 |
|  | **n-RFI** | 12 | 0.989 ± 0.003 | 0.955 ± 0.007 | 31,429 ± 8,034 | 1,436 ± 118 | 9,926 ± 106 | 1,079 ± 84 | 8,760 ± 451 | 713 ± 37 |
| **Solid** | **p-RFI** | 15 | 0.989 ± 0.003 | 0.963 ± 0.009 | 28,964 ± 8,102 | 1,231 ± 173 | 9,923 ± 98 | 936 ± 153 | 8,597 ± 474 | 624 ± 74 |
|  | **n-RFI** | 12 | 0.987 ± 0.006 | 0.959 ± 0.010 | 27,754 ± 9,581 | 1,245 ± 201 | 10,000 ± 120 | 995 ± 118 | 8,845 ± 500 | 666 ± 51 |
| **ARCHAEA** |  |  |  |  |  |  |  |  |  |  |
| **Liquid** | **p-RFI** | 14 | 0.998 ± 0.001 | 0.993 ± 0.003 | 3,898 ± 1,728 | 38 ± 9 | 1,164 ± 4 | 29 ± 4 | 1,144 ± 30 | 22 ± 2 |
|  | **n-RFI** | 12 | 0.998 ± 0.002 | 0.991 ± 0.003 | 4,575 ± 3,116 | 37 ± 9 | 1,164 ± 4 | 28 ± 5 | 1,152 ± 9 | 22 ± 3 |
| **Solid** | **p-RFI** | 14 | 0.998 ± 0.001 | 0.993 ± 0.002 | 5,379 ± 1,646 | 41 ± 7 | 1,163 ± 2 | 27 ± 4 | 1,145 ± 20 | 21 ± 2 |
|  | **n-RFI** | 12 | 0.999 ± 0.001 | 0.991 ± 0.003 | 5,519 ± 1,428 | 44 ± 7 | 1,164 ± 2 | 31 ± 4 | 1,153 ± 9 | 24 ± 2 |
| **FUNGI** |  |  |  |  |  |  |  |  |  |  |
| **Liquid** | **p-RFI** | 15 | 0.945 ± 0.017 | 0.975 ± 0.012 | 14,594 ± 10,990 | 874 ± 606 | 1,616 ± 48 | 89 ± 48 | 1,211 ± 363 | 17 ± 3 |
|  | **n-RFI** | 12 | 0.955 ± 0.016 | 0.976 ± 0.009 | 21,897 ± 12,586 | 1,083 ± 428 | 1,604 ± 22 | 78 ± 27 | 1,328 ± 211 | 17 ± 2 |
| **Solid** | **p-RFI** | 15 | 0.950 ± 0.023 | 0.979 ± 0.011 | 25,292 ± 12,284 | 1,298 ± 297 | 1,588 ± 43 | 64 ± 24 | 1,201 ± 393 | 16 ± 5 |
|  | **n-RFI** | 11 | 0.946 ± 0.016 | 0.977 ± 0.006 | 16,660 ± 5,475 | 1,110 ± 444 | 1,587 ± 34 | 71 ± 16 | 1,285 ± 234 | 19 ± 3 |

^a^ Values represent mean and standard deviation

^b^Reads and OTUs that were detected in at least half of the steers in each feed efficiency group (at least 7 animals to p-RFI and 6 animals to n-RFI)

**a**

**b**

**Supplementary Fig. S1** Ruminal bacterial composition at the phyla level of rumen liquids (a) and rumen solids (b) samples. Each bar represents a different steer and the mean composition of the p-RFI and n-RFI bacterial community is shown in the center of the figure. “Other” corresponds to the sum of phyla that showed relative abundance < 0.1 %

**a**

**b**

**Supplementary Fig. S2** Ruminal bacterial composition at the family level of rumen liquids (a) and rumen solids (b) samples. Each bar represents a different steer and the mean composition of the p-RFI and n-RFI bacterial community is shown in the center of the figure. “Other” corresponds to the sum of phyla that showed relative abundance < 0.2 %


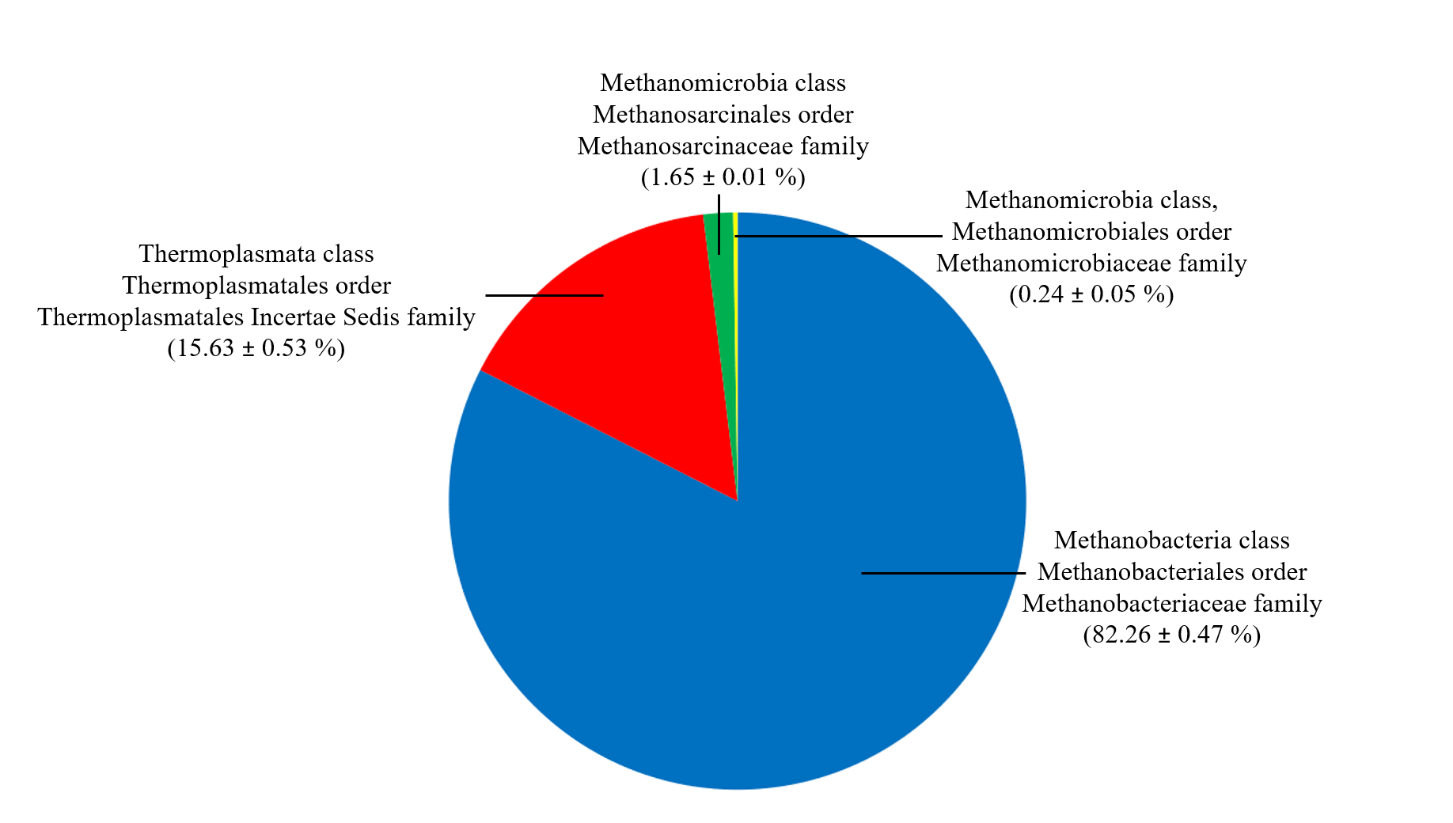


**Supplementary Fig. S3** Composition of the ruminal archaea at the class, order and family levels

**a**

**b**

**Supplementary Fig. S4** Ruminal archaeal composition at the genus level of rumen liquids (a) and rumen solids (b) samples. Each bar represents a different p-RFI or n-RFI steer

**Supplementary Table S3** Ruminal fungal composition (%) at genus-level according to RFI group^a^

| **Genus** | | **Rumen liquid** | | **Rumen solid** | | | |
| --- | --- | --- | --- | --- | --- | --- | --- |
|  |  | **p-RFI** | **n-RFI** | **p-RFI** | | **n-RFI** | |
| *Caecomyces* | 39.45 ± 20.06 | | 35.63 ± 14.10 | | 67.24 ± 19.85 | | 64.13 ± 9.23 |
| *Piromyces* | 15.11 ± 9.10 | | 18.78 ± 11.52 | | 7.59 ± 5.00 | | 8.81 ± 7.37 |
| *Orpinomyces* | 18.07 ± 15.04 | | 16.40 ± 17.21 | | 4.85 ± 5.61 | | 5.44 ± 5.76 |
| *Cyllamyces* | 1.09 ± 3.61 | | 0.42 ± 0.64 | | 3.96 ± 4.22 | | 3.03 ± 1.67 |
| *Neocallimastix* | 1.39 ± 0.83 | | 1.41 ± 0.54 | | 0.81 ± 0.43 | | 1.04 ± 0.71 |
| *Anaeromyces* | 0.76 ± 1.08 | | 0.40 ± 0.5 | | 0.28 ± 0.27 | | 0.24 ± 0.30 |
| *Buwchfawromyces* | 0.04 ± 0.11 | | 0.13 ± 0.17 | | 0.03 ± 0.05 | | 0.14 ± 0.15 |
| Unclassified | 24.09 ± 10.28 | | 26.83 ± 10.41 | | 15.24 ± 4.39 | | 17.17 ± 4.71 |

^a^Data represents the relative abundance mean ± standard deviation

**Supplementary Table S4** Relative abundance (%) of the functional categories predicted for the bacterial microbiota in liquid and solid ruminal fractions of Nellore steers showing high (n-RFI) and low (p-RFI) feed efficiency

| **Functional category** | **Rumen liquid** | | | **Rumen solid** | | |
| --- | --- | --- | --- | --- | --- | --- |
|  | **p-RFI** | **n-RFI** | ***P*-values^a^** | **p-RFI** | **n-RFI** | ***P*-values^a^** |
| Transporters | 4.357 | 4.393 | 0.695 | 4.216 | 4.242 | 0.859 |
| Two-component system | 3.729 | 3.813 | 0.118 | 3.709 | 3.750 | 0.637 |
| DNA repair and recombination proteins | 3.091 | 3.066 | 0.088 | 3.051 | 3.040 | 0.598 |
| ABC transporters | 2.749 | 2.775 | 0.691 | 2.658 | 2.692 | 0.775 |
| Purine metabolism | 2.583 | 2.552 | 0.072 | 2.596 | 2.575 | 0.399 |
| Ribosome | 2.476 | 2.452 | 0.168 | 2.483 | 2.458 | 0.531 |
| Peptidases | 2.158 | 2.156 | 0.813 | 2.162 | 2.165 | 0.863 |
| Pyrimidine metabolism | 2.117 | 2.096 | 0.210 | 2.136 | 2.116 | 0.496 |
| Ribosome biogenesis | 2.046 | 2.031 | 0.125 | 2.032 | 2.016 | 0.273 |
| Transcription factors | 1.896 | 1.901 | 0.884 | 1.835 | 1.833 | 0.976 |
| Amino acid related enzymes | 1.871 | 1.865 | 0.591 | 1.879 | 1.876 | 0.903 |
| Chromosome | 1.694 | 1.691 | 0.854 | 1.689 | 1.701 | 0.646 |
| General function prediction only | 1.691 | 1.688 | 0.781 | 1.691 | 1.691 | 0.978 |
| Amino sugar and nucleotide sugar metabolism | 1.442 | 1.446 | 0.703 | 1.464 | 1.463 | 0.936 |
| Other ion-coupled transporters | 1.392 | 1.377 | 0.364 | 1.401 | 1.402 | 0.966 |
| Aminoacyl-trna biosynthesis | 1.374 | 1.366 | 0.208 | 1.363 | 1.359 | 0.756 |
| Starch and sucrose metabolism | 1.350 | 1.334 | 0.388 | 1.408 | 1.378 | 0.303 |
| Protein kinases | 1.314 | 1.365 | 0.081 | 1.338 | 1.364 | 0.413 |
| Transcription machinery | 1.282 | 1.283 | 0.919 | 1.306 | 1.312 | 0.749 |
| Alanine, aspartate and glutamate metabolism | 1.232 | 1.231 | 0.891 | 1.235 | 1.230 | 0.679 |
| Arginine and proline metabolism | 1.203 | 1.196 | 0.190 | 1.214 | 1.217 | 0.801 |
| DNA replication proteins | 1.193 | 1.178 | 0.095 | 1.178 | 1.171 | 0.572 |
| Methane metabolism | 1.171 | 1.173 | 0.805 | 1.149 | 1.159 | 0.501 |
| Chaperones and folding catalysts | 1.152 | 1.151 | 0.894 | 1.166 | 1.167 | 0.959 |
| Phenylalanine, tyrosine and tryptophan biosynthesis | 1.027 | 1.026 | 0.891 | 1.028 | 1.026 | 0.908 |
| Cysteine and methionine metabolism | 1.002 | 1.001 | 0.937 | 0.986 | 0.996 | 0.796 |
| Carbon fixation pathways in prokaryotes | 1.000 | 0.999 | 0.859 | 0.989 | 0.994 | 0.453 |
| Lysine biosynthesis | 0.988 | 0.984 | 0.466 | 0.991 | 0.994 | 0.644 |
| Homologous recombination | 0.978 | 0.968 | 0.061 | 0.965 | 0.959 | 0.517 |
| Glycine, serine and threonine metabolism | 0.955 | 0.957 | 0.850 | 0.960 | 0.961 | 0.946 |
| Pores ion channels | 0.946 | 0.937 | 0.848 | 1.031 | 1.026 | 0.940 |
| Bacterial motility proteins | 0.938 | 0.968 | 0.404 | 0.958 | 0.967 | 0.910 |
| Peptidoglycan biosynthesis | 0.927 | 0.923 | 0.355 | 0.917 | 0.915 | 0.763 |
| Lipid biosynthesis proteins | 0.913 | 0.920 | 0.461 | 0.896 | 0.892 | 0.775 |
| Translation proteins | 0.906 | 0.899 | 0.186 | 0.888 | 0.879 | 0.297 |
| Pyruvate metabolism | 0.855 | 0.860 | 0.549 | 0.848 | 0.850 | 0.837 |
| Mismatch repair | 0.839 | 0.832 | 0.169 | 0.821 | 0.815 | 0.281 |
| Oxidative phosphorylation | 0.825 | 0.823 | 0.741 | 0.829 | 0.835 | 0.601 |
| Energy metabolism | 0.793 | 0.787 | 0.421 | 0.801 | 0.798 | 0.780 |
| Glycolysis / gluconeogenesis | 0.768 | 0.771 | 0.723 | 0.758 | 0.756 | 0.861 |
| Secretion system | 0.758 | 0.761 | 0.755 | 0.760 | 0.759 | 0.903 |
| Cell cycle - Caulobacter | 0.754 | 0.752 | 0.753 | 0.747 | 0.745 | 0.640 |
| Pentose phosphate pathway | 0.752 | 0.748 | 0.301 | 0.752 | 0.748 | 0.601 |
| One carbon pool by folate | 0.724 | 0.727 | 0.677 | 0.720 | 0.739 | 0.292 |
| Pantothenate and coa biosynthesis | 0.702 | 0.702 | 0.997 | 0.701 | 0.704 | 0.796 |
| Nitrogen metabolism | 0.697 | 0.695 | 0.673 | 0.700 | 0.696 | 0.418 |
| Butanoate metabolism | 0.690 | 0.690 | 0.963 | 0.685 | 0.686 | 0.892 |
| Pentose and glucuronate interconversions | 0.686 | 0.675 | 0.510 | 0.748 | 0.738 | 0.739 |
| Valine, leucine and isoleucine biosynthesis | 0.683 | 0.683 | 0.996 | 0.686 | 0.687 | 0.828 |
| DNA replication | 0.681 | 0.670 | 0.062 | 0.665 | 0.661 | 0.523 |
| Histidine metabolism | 0.659 | 0.657 | 0.618 | 0.662 | 0.659 | 0.642 |
| Fructose and mannose metabolism | 0.626 | 0.636 | 0.471 | 0.663 | 0.672 | 0.671 |
| Bacterial chemotaxis | 0.625 | 0.648 | 0.284 | 0.628 | 0.641 | 0.799 |
| Membrane and intracellular structural molecules | 0.618 | 0.617 | 0.924 | 0.625 | 0.630 | 0.545 |
| Nicotinate and nicotinamide metabolism | 0.615 | 0.611 | 0.499 | 0.628 | 0.627 | 0.917 |
| Porphyrin and chlorophyll metabolism | 0.610 | 0.618 | 0.598 | 0.596 | 0.609 | 0.358 |
| Fatty acid biosynthesis | 0.605 | 0.619 | 0.091 | 0.600 | 0.604 | 0.630 |
| Terpenoid backbone biosynthesis | 0.601 | 0.599 | 0.567 | 0.606 | 0.604 | 0.793 |
| Galactose metabolism | 0.584 | 0.584 | 0.984 | 0.608 | 0.606 | 0.924 |
| Translation factors | 0.566 | 0.561 | 0.093 | 0.564 | 0.560 | 0.480 |
| Lipopolysaccharide biosynthesis proteins | 0.556 | 0.568 | 0.576 | 0.602 | 0.614 | 0.582 |
| Propanoate metabolism | 0.548 | 0.554 | 0.350 | 0.534 | 0.536 | 0.759 |
| RNA degradation | 0.547 | 0.539 | 0.079 | 0.539 | 0.533 | 0.288 |
| Glyoxylate and dicarboxylate metabolism | 0.541 | 0.538 | 0.507 | 0.538 | 0.534 | 0.366 |
| Protein folding and associated processing | 0.533 | 0.533 | 0.934 | 0.533 | 0.530 | 0.662 |
| Citrate cycle (TCA cycle) | 0.523 | 0.520 | 0.663 | 0.516 | 0.519 | 0.818 |
| Signal transduction mechanisms | 0.518 | 0.533 | 0.158 | 0.541 | 0.551 | 0.318 |
| Carbon fixation in photosynthetic organisms | 0.517 | 0.516 | 0.889 | 0.517 | 0.518 | 0.957 |
| Glycerophospholipid metabolism | 0.508 | 0.504 | 0.405 | 0.497 | 0.492 | 0.306 |
| Glycosyltransferases | 0.499 | 0.493 | 0.566 | 0.516 | 0.513 | 0.834 |
| Streptomycin biosynthesis | 0.485 | 0.492 | 0.416 | 0.505 | 0.508 | 0.820 |
| Selenocompound metabolism | 0.477 | 0.488 | 0.308 | 0.467 | 0.484 | 0.478 |
| Function unknown | 0.464 | 0.462 | 0.743 | 0.448 | 0.451 | 0.698 |
| Bacterial secretion system | 0.459 | 0.456 | 0.446 | 0.465 | 0.461 | 0.613 |
| Protein export | 0.446 | 0.442 | 0.195 | 0.445 | 0.441 | 0.380 |
| Sporulation | 0.443 | 0.460 | 0.223 | 0.438 | 0.453 | 0.398 |
| Drug metabolism - other enzymes | 0.429 | 0.422 | 0.233 | 0.440 | 0.432 | 0.291 |
| Cyanoamino acid metabolism | 0.419 | 0.401 | 0.202 | 0.450 | 0.434 | 0.284 |
| Thiamine metabolism | 0.400 | 0.398 | 0.694 | 0.391 | 0.391 | 0.983 |
| Nucleotide excision repair | 0.367 | 0.364 | 0.166 | 0.364 | 0.364 | 0.829 |
| Nucleotide metabolism | 0.366 | 0.357 | 0.382 | 0.340 | 0.331 | 0.391 |
| Sulfur metabolism | 0.362 | 0.358 | 0.234 | 0.358 | 0.353 | 0.301 |
| Carbohydrate metabolism | 0.358 | 0.370 | 0.162 | 0.353 | 0.357 | 0.595 |
| Cytoskeleton proteins | 0.357 | 0.355 | 0.697 | 0.360 | 0.360 | 0.978 |
| Flagellar assembly | 0.342 | 0.349 | 0.637 | 0.362 | 0.361 | 0.988 |
| Lipopolysaccharide biosynthesis | 0.337 | 0.342 | 0.750 | 0.365 | 0.371 | 0.720 |
| Photosynthesis | 0.336 | 0.335 | 0.757 | 0.329 | 0.324 | 0.501 |
| Photosynthesis proteins | 0.336 | 0.335 | 0.757 | 0.329 | 0.324 | 0.501 |
| Base excision repair | 0.333 | 0.332 | 0.726 | 0.327 | 0.328 | 0.429 |
| Replication, recombination and repair proteins | 0.328 | 0.326 | 0.236 | 0.323 | 0.319 | 0.296 |
| Peroxisome | 0.325 | 0.321 | 0.524 | 0.321 | 0.318 | 0.608 |
| Other transporters | 0.302 | 0.302 | 0.969 | 0.305 | 0.307 | 0.718 |
| Polyketide sugar unit biosynthesis | 0.301 | 0.306 | 0.532 | 0.317 | 0.321 | 0.680 |
| Fatty acid metabolism | 0.300 | 0.297 | 0.223 | 0.291 | 0.285 | 0.185 |
| Glycerolipid metabolism | 0.281 | 0.284 | 0.665 | 0.269 | 0.268 | 0.876 |
| Folate biosynthesis | 0.261 | 0.258 | 0.421 | 0.254 | 0.252 | 0.780 |
| Vitamin B6 metabolism | 0.261 | 0.259 | 0.661 | 0.267 | 0.266 | 0.881 |
| Phenylpropanoid biosynthesis | 0.256 | 0.240 | 0.224 | 0.283 | 0.270 | 0.299 |
| Phenylalanine metabolism | 0.242 | 0.245 | 0.424 | 0.241 | 0.242 | 0.907 |
| PPAR signaling pathway | 0.238 | 0.235 | 0.466 | 0.229 | 0.224 | 0.496 |
| Benzoate degradation | 0.234 | 0.233 | 0.803 | 0.223 | 0.222 | 0.777 |
| Valine, leucine and isoleucine degradation | 0.232 | 0.232 | 0.978 | 0.232 | 0.232 | 0.973 |
| Prenyltransferases | 0.230 | 0.228 | 0.811 | 0.242 | 0.243 | 0.932 |
| C5-Branched dibasic acid metabolism | 0.211 | 0.209 | 0.492 | 0.214 | 0.212 | 0.779 |
| Riboflavin metabolism | 0.206 | 0.208 | 0.584 | 0.208 | 0.212 | 0.265 |
| Adipocytokine signaling pathway | 0.200 | 0.198 | 0.499 | 0.194 | 0.190 | 0.622 |
| Plant-pathogen interaction | 0.198 | 0.197 | 0.908 | 0.203 | 0.201 | 0.674 |
| RNA polymerase | 0.193 | 0.191 | 0.127 | 0.190 | 0.189 | 0.430 |
| Novobiocin biosynthesis | 0.192 | 0.192 | 0.832 | 0.190 | 0.190 | 0.827 |
| Biosynthesis of unsaturated fatty acids | 0.188 | 0.191 | 0.269 | 0.188 | 0.188 | 0.937 |
| Glutathione metabolism | 0.184 | 0.184 | 0.969 | 0.188 | 0.188 | 0.914 |
| D-Glutamine and D-glutamate metabolism | 0.182 | 0.181 | 0.227 | 0.181 | 0.180 | 0.572 |
| Tyrosine metabolism | 0.179 | 0.180 | 0.712 | 0.177 | 0.177 | 0.888 |
| Ubiquinone and other terpenoid-quinone biosynthesis | 0.175 | 0.172 | 0.643 | 0.195 | 0.195 | 0.943 |
| Phosphotransferase system (PTS) | 0.174 | 0.163 | 0.082 | 0.165 | 0.153 | 0.088 |
| Beta-Alanine metabolism | 0.170 | 0.173 | 0.397 | 0.175 | 0.178 | 0.352 |
| Taurine and hypotaurine metabolism | 0.166 | 0.165 | 0.752 | 0.170 | 0.170 | 0.941 |
| Vibrio cholerae pathogenic cycle | 0.166 | 0.164 | 0.712 | 0.169 | 0.166 | 0.484 |
| Sulfur relay system | 0.162 | 0.160 | 0.398 | 0.160 | 0.158 | 0.667 |
| Lipid metabolism | 0.154 | 0.155 | 0.617 | 0.153 | 0.152 | 0.829 |
| Tropane, piperidine and pyridine alkaloid biosynthesis | 0.153 | 0.154 | 0.779 | 0.153 | 0.153 | 0.894 |
| Restriction enzyme | 0.153 | 0.150 | 0.601 | 0.157 | 0.154 | 0.649 |
| Ascorbate and aldarate metabolism | 0.138 | 0.131 | 0.162 | 0.147 | 0.142 | 0.393 |
| Tuberculosis | 0.137 | 0.137 | 0.991 | 0.129 | 0.129 | 0.946 |
| Amino acid metabolism | 0.131 | 0.137 | 0.142 | 0.132 | 0.138 | 0.260 |
| D-Alanine metabolism | 0.129 | 0.128 | 0.262 | 0.126 | 0.126 | 0.843 |
| Epithelial cell signaling in Helicobacter pylori infection | 0.128 | 0.127 | 0.339 | 0.126 | 0.125 | 0.657 |
| Inositol phosphate metabolism | 0.116 | 0.120 | 0.175 | 0.117 | 0.118 | 0.822 |
| Biotin metabolism | 0.112 | 0.112 | 0.794 | 0.106 | 0.106 | 0.938 |
| Biosynthesis of vancomycin group antibiotics | 0.112 | 0.114 | 0.666 | 0.117 | 0.118 | 0.757 |
| Cell division | 0.109 | 0.109 | 0.751 | 0.109 | 0.109 | 0.965 |
| Tryptophan metabolism | 0.107 | 0.105 | 0.486 | 0.107 | 0.107 | 0.999 |
| Cell motility and secretion | 0.107 | 0.106 | 0.537 | 0.105 | 0.104 | 0.701 |
| Chloroalkane and chloroalkene degradation | 0.100 | 0.101 | 0.868 | 0.109 | 0.109 | 0.950 |
| Others^b^ | 4.112 | 4.121 | > 0.05 | 4.108 | 4.112 | > 0.05 |

^a^White’s non-parametric t-test comparing the means of the feed efficiency groups

^b^Correspond to the sum of functional categories that showed relative abundance < 0.1 %

**Supplementary Table S5** Ruminal fermentation variables of Nellore steers according to their RFI group

| **Item** | **p-RFI** | | **n-RFI** | | ***P*-value^a^** |
| --- | --- | --- | --- | --- | --- |
|  | **Mean** | **SEM** | **Mean** | **SEM** |  |
| **Acetic acid (%)** | 69.37 | 0.47 | 69.34 | 0.62 | 0.972 |
| **Propionic acid (%)** | 14.88 | 0.54 | 14.05 | 0.52 | 0.275 |
| **Butyric acid (%)** | 6.93 | 0.26 | 6.83 | 0.29 | 0.789 |
| **Isobutyric acid (%)** | 3.54 | 0.15 | 4.05 | 0.33 | 0.175 |
| **Valeric acid (%)** | 0.97 | 0.08 | 0.98 | 0.04 | 0.953 |
| **Isovaleric acid (%)** | 3.72 | 0.19 | 4.02 | 0.36 | 0.467 |
| **Succinic acid (%)** | 0.59 | 0.08 | 0.73 | 0.13 | 0.352 |
| **A/P ratio^b^** | 4.75 | 0.19 | 5.02 | 0.21 | 0.362 |
| **Total VFA^c^ (mmol/L)** | 53.85 | 4.64 | 49.24 | 4.18 | 0.468 |
| **Ammonia (mg/L)** | 7.92 | 0.70 | 7.43 | 0.82 | 0.648 |
| **pH** | 6.58 | 0.07 | 6.68 | 0.09 | 0.382 |

The values represent the mean and standard error of mean (SEM)

^a^t-test

^b^Acetic to propionic acid ratio

^c^Total concentration of volatile fatty acids

**Supplementary Table S6** Relative abundance of the most abundant bacterial OTUs showing positive correlation with ruminal fermentation parameters^a^ in p-RFI steers

| **Parameter** | **Liquid** | | | | **Solid** | | | |
| --- | --- | --- | --- | --- | --- | --- | --- | --- |
|  | **OTU** | **Taxonomy^b^** | **Relative abundance (%)** | **SEM^c^** | **OTU** | **Taxonomy^b^** | **Relative abundance (%)** | **SEM^c^** |
| **Ace** | **Otu00064** | *Prevotella* 1 | 0.468 | 0.198 | **Otu00028** | Rikenellaceae RC9 gut group | 0.735 | 0.126 |
|  | **Otu00057** | Bacteroidales BS11 gut group ge | 0.461 | 0.181 | **Otu00031** | *Prevotella* 1 | 0.619 | 0.307 |
|  | **Otu00031** | *Prevotella* 1 | 0.449 | 0.135 | **Otu00016** | *Prevotella* 1 | 0.455 | 0.108 |
|  | **Otu00197** | Rikenellaceae RC9 gut group | 0.170 | 0.079 | **Otu00086** | Lachnospiraceae AC2044 group | 0.455 | 0.054 |
|  | **Otu00138** | Prevotellaceae UCG-001 | 0.139 | 0.047 | **Otu00095** | Rikenellaceae RC9 gut group | 0.407 | 0.071 |
| **Prop** | **Otu00003** | Rikenellaceae RC9 gut group | 1.951 | 0.399 | **Otu00003** | Rikenellaceae RC9 gut group | 1.752 | 0.328 |
|  | **Otu00044** | Christensenellaceae R-7 group | 0.629 | 0.550 | **Otu00078** | Lachnospiraceae ge | 0.331 | 0.065 |
|  | **Otu00048** | Bacteroidales BS11 gut group ge | 0.448 | 0.068 | **Otu00066** | *Phocaeicola* | 0.293 | 0.101 |
|  | **Otu00198** | Planctomycetaceae p-1088-a5 gut group | 0.097 | 0.026 | **Otu00177** | *Fibrobacter* | 0.176 | 0.043 |
|  | **Otu00214** | *Saccharofermentans* | 0.094 | 0.026 | **Otu00258** | Prevotellaceae UCG-004 | 0.104 | 0.045 |
| **But** | **Otu00172** | *Succiniclasticum* | 0.105 | 0.037 | **Otu00187** | *Acetitomaculum* | 0.150 | 0.053 |
|  | **Otu00187** | *Acetitomaculum* | 0.059 | 0.019 | **Otu00124** | Ruminococcaceae NK4A214 group | 0.133 | 0.025 |
|  | **Otu00386** | Bifidobacteriaceae uncultured | 0.054 | 0.025 | **Otu00181** | Clostridiales Family XIII unclassified | 0.132 | 0.021 |
|  | **Otu00312** | Christensenellaceae R-7 group | 0.051 | 0.009 | **Otu00071** | Verrucomicrobia WCHB1-41 ge | 0.126 | 0.018 |
|  | **Otu00376** | Rikenellaceae RC9 gut group | 0.034 | 0.011 | **Otu00172** | *Succiniclasticum* | 0.093 | 0.037 |
| **Isobut** | **Otu00006** | *Butyrivibrio* 2 | 0.588 | 0.137 | **Otu00015** | Rikenellaceae RC9 gut group | 1.026 | 0.299 |
|  | **Otu00015** | Rikenellaceae RC9 gut group | 0.464 | 0.151 | **Otu00023** | *Papillibacter* | 0.955 | 0.062 |
|  | **Otu00057** | Bacteroidales BS11 gut group ge | 0.461 | 0.181 | **Otu00021** | Christensenellaceae R-7 group | 0.682 | 0.336 |
|  | **Otu00013** | *Pseudobutyrivibrio* | 0.450 | 0.064 | **Otu00096** | *Prevotella* 1 | 0.309 | 0.131 |
|  | **Otu00096** | *Prevotella* 1 | 0.288 | 0.121 | **Otu00072** | Ruminococcaceae ge | 0.192 | 0.044 |
| **Suc** | **Otu00011** | *Prevotella* 1 | 1.826 | 0.626 | **Otu00011** | *Prevotella* 1 | 0.506 | 0.094 |
|  | **Otu00112** | Bacteroidales UCG-001 ge | 0.198 | 0.034 | **Otu00082** | *Moryella* | 0.288 | 0.026 |
|  | **Otu00150** | Ruminococcaceae UCG-005 | 0.162 | 0.028 | **Otu00187** | *Acetitomaculum* | 0.150 | 0.053 |
|  | **Otu00180** | Ruminococcaceae UCG-002 | 0.130 | 0.021 | **Otu00124** | Ruminococcaceae NK4A214 group | 0.133 | 0.025 |
|  | **Otu00245** | Christensenellaceae R-7 group | 0.087 | 0.023 | **Otu00320** | Bacteroidetes BD2-2 ge | 0.112 | 0.071 |
| **Val** | **Otu00047** | Ruminococcaceae NK4A214 group | 0.442 | 0.124 | **Otu00047** | Ruminococcaceae NK4A214 group | 0.426 | 0.083 |
|  | **Otu00035** | *Mogibacterium* | 0.415 | 0.152 | **Otu00081** | Lachnospiraceae NK3A20 group | 0.281 | 0.039 |
|  | **Otu00116** | Lachnospiraceae ND3007 group | 0.189 | 0.061 | **Otu00183** | *Ruminococcus* 1 | 0.144 | 0.021 |
|  | **Otu00181** | Clostridiales Family XIII unclassified | 0.143 | 0.034 | **Otu00181** | Clostridiales Family XIII unclassified | 0.132 | 0.021 |
|  | **Otu00065** | Rikenellaceae RC9 gut group | 0.128 | 0.043 | **Otu00342** | Chloroflexi unclassified | 0.079 | 0.015 |
| **Isoval** | **Otu00015** | Rikenellaceae RC9 gut group | 0.464 | 0.151 | **Otu00015** | Rikenellaceae RC9 gut group | 1.026 | 0.299 |
|  | **Otu00098** | Prevotellaceae UCG-003 | 0.147 | 0.031 | **Otu00036** | Rikenellaceae U29-B03 | 0.511 | 0.175 |
|  | **Otu00164** | Veillonellaceae UCG-001 | 0.135 | 0.030 | **Otu00103** | Ruminococcaceae NK4A214 group | 0.295 | 0.037 |
|  | **Otu00245** | Christensenellaceae R-7 group | 0.087 | 0.023 | **Otu00184** | Lachnospiraceae XPB1014 group | 0.174 | 0.051 |
|  | **Otu00274** | Prevotellaceae UCG-003 | 0.083 | 0.022 | **Otu00183** | *Ruminococcus* 1 | 0.144 | 0.021 |
| **Ace/Prop** | **Otu00064** | *Prevotella* 1 | 0.468 | 0.198 | **Otu00015** | Rikenellaceae RC9 gut group | 1.026 | 0.299 |
|  | **Otu00015** | Rikenellaceae RC9 gut group | 0.464 | 0.151 | **Otu00011** | *Prevotella* 1 | 0.506 | 0.094 |
|  | **Otu00057** | Bacteroidales BS11 gut group ge | 0.461 | 0.181 | **Otu00095** | Rikenellaceae RC9 gut group | 0.407 | 0.071 |
|  | **Otu00197** | Rikenellaceae RC9 gut group | 0.170 | 0.079 | **Otu00074** | Lachnospiraceae XPB1014 group | 0.358 | 0.068 |
|  | **Otu00207** | *Spirochaeta* 2 | 0.114 | 0.019 | **Otu00064** | *Prevotella* 1 | 0.350 | 0.107 |
| **VFAt** | **Otu00038** | *Papillibacter* | 0.328 | 0.047 | **Otu00025** | Bacteroidales BS11 gut group ge | 0.136 | 0.039 |
|  | **Otu00210** | Bacteroidales BS11 gut group ge | 0.168 | 0.138 | **Otu00210** | Bacteroidales BS11 gut group ge | 0.077 | 0.059 |
|  | **Otu00234** | Ruminococcaceae ge | 0.089 | 0.023 | **Otu00327** | *Treponema* 2 | 0.076 | 0.020 |
|  | **Otu00258** | Prevotellaceae UCG-004 | 0.082 | 0.043 | **Otu00234** | Ruminococcaceae ge | 0.069 | 0.019 |
|  | **Otu00253** | Lachnospiraceae unclassified | 0.058 | 0.023 | **Otu00220** | Bacteroidales BS11 gut group ge | 0.061 | 0.035 |
| **pH** | **Otu00064** | *Prevotella* 1 | 0.468 | 0.198 | **Otu00028** | Rikenellaceae RC9 gut group | 0.735 | 0.126 |
|  | **Otu00023** | *Papillibacter* | 0.372 | 0.030 | **Otu00011** | *Prevotella* 1 | 0.506 | 0.094 |
|  | **Otu00197** | Rikenellaceae RC9 gut group | 0.170 | 0.079 | **Otu00095** | Rikenellaceae RC9 gut group | 0.407 | 0.071 |
|  | **Otu00190** | Rikenellaceae RC9 gut group | 0.120 | 0.046 | **Otu00074** | Lachnospiraceae XPB1014 group | 0.358 | 0.068 |
|  | **Otu00236** | Victivallales vadinBE97 ge | 0.112 | 0.017 | **Otu00072** | Ruminococcaceae ge | 0.192 | 0.044 |
| **NH** | **Otu00048** | Bacteroidales BS11 gut group ge | 0.448 | 0.068 | **Otu00003** | Rikenellaceae RC9 gut group | 1.752 | 0.328 |
|  | **Otu00191** | Ruminococcaceae NK4A214 group | 0.117 | 0.030 | **Otu00005** | *Succiniclasticum* | 1.541 | 0.175 |
|  | **Otu00239** | Ruminococcaceae ge | 0.081 | 0.014 | **Otu00019** | Prevotellaceae NK3B31 group | 1.023 | 0.805 |
|  | **Otu00316** | Anaerolineaceae unclassified | 0.041 | 0.009 | **Otu00041** | *Saccharofermentans* | 0.677 | 0.268 |
|  | **Otu00302** | Ruminococcaceae UCG-010 | 0.033 | 0.008 | **Otu00052** | Clostridiales unclassified | 0.394 | 0.108 |

^a^Fermentation parameters: molar proportion of acetic, Ace; propionic, Prop; succinic, Suc; butyric, But; isobutyric, Isobut; valeric, val; and isovaleric acids, Isoval; acetate-to-propionate ratio, Ace/Prop; total concentration of these volatile fats acids, VFAt; ammonia concentration, NH_3_; and pH

^b^Taxonomy for each OTU is given at the highest classifiable level

^c^Standard error of mean (SEM)

**Supplementary Table S7** Relative abundance of the most abundant bacterial OTUs showing positive correlation with ruminal fermentation parameters^a^ in n-RFI steers

| **Parameter** | **Liquid** | | | | **Solid** | | | |
| --- | --- | --- | --- | --- | --- | --- | --- | --- |
|  | **OTU** | **Taxonomy^b^** | **Relative abundance (%)** | **SEM^c^** | **OTU** | **Taxonomy^b^** | **Relative abundance (%)** | **SEM^c^** |
| **Ace** | **Otu00156** | Ruminococcaceae UCG-005 | 0.204 | 0.086 | **Otu00088** | *Fibrobacter* | 0.344 | 0.141 |
|  | **Otu00154** | Rikenellaceae RC9 gut group | 0.130 | 0.033 | **Otu00101** | Veillonellaceae UCG-001 | 0.192 | 0.025 |
|  | **Otu00120** | *Butyrivibrio* 2 | 0.096 | 0.035 | **Otu00131** | Christensenellaceae R-7 group | 0.167 | 0.080 |
|  | **Otu00269** | *Selenomonas* 1 | 0.070 | 0.012 | **Otu00276** | *Lachnoclostridium* 10 | 0.155 | 0.107 |
|  | **Otu00280** | *Treponema* 2 | 0.060 | 0.014 | **Otu00251** | Bacteroidetes BD2-2 ge | 0.118 | 0.023 |
| **Prop** | **Otu00028** | Rikenellaceae RC9 gut group | 0.380 | 0.082 | **Otu00184** | Lachnospiraceae XPB1014 group | 0.208 | 0.047 |
|  | **Otu00326** | Prevotellaceae UCG-003 | 0.182 | 0.094 | **Otu00109** | Prevotellaceae UCG-004 | 0.168 | 0.071 |
|  | **Otu00205** | Verrucomicrobia WCHB1-41 ge | 0.148 | 0.063 | **Otu00310** | Clostridiales Family XIII AD3011 group | 0.093 | 0.037 |
|  | **Otu00109** | Prevotellaceae UCG-004 | 0.137 | 0.056 | **Otu00375** | Lachnospiraceae unclassified | 0.075 | 0.016 |
|  | **Otu00159** | Ruminococcaceae NK4A214 group | 0.110 | 0.017 | **Otu00347** | Ruminococcaceae UCG-002 | 0.068 | 0.018 |
| **But** | **Otu00195** | Ruminococcaceae UCG-014 | 0.121 | 0.040 | **Otu00026** | Lachnospiraceae NK3A20 group | 0.694 | 0.183 |
|  | **Otu00220** | Bacteroidales BS11 gut group ge | 0.084 | 0.031 | **Otu00091** | Ruminococcaceae NK4A214 group | 0.277 | 0.048 |
|  | **Otu00341** | Ruminococcaceae UCG-010 | 0.076 | 0.017 | **Otu00183** | *Ruminococcus* 1 | 0.221 | 0.102 |
|  | **Otu00238** | Bacteroidales BS11 gut group ge | 0.052 | 0.026 | **Otu00097** | Clostridiales Family XIII AD3011 group | 0.212 | 0.023 |
|  | **Otu01121** | Bacteroidales RF16 group ge | 0.024 | 0.006 | **Otu00226** | *Prevotella* 1 | 0.147 | 0.019 |
| **Isobut** | **Otu00043** | Christensenellaceae R-7 group | 0.373 | 0.171 | **Otu00012** | *Prevotella* 1 | 0.928 | 0.188 |
|  | **Otu00162** | Bacteroidales BS11 gut group ge | 0.277 | 0.223 | **Otu00033** | Lachnospiraceae unclassified | 0.656 | 0.166 |
|  | **Otu00033** | Lachnospiraceae unclassified | 0.245 | 0.081 | **Otu00163** | Lachnospiraceae probable genus 10 | 0.289 | 0.136 |
|  | **Otu00063** | *Prevotella* 1 | 0.147 | 0.037 | **Otu00155** | *Ruminococcus* 1 | 0.232 | 0.125 |
|  | **Otu00119** | Anaerolineaceae uncultured | 0.120 | 0.022 | **Otu00072** | Ruminococcaceae ge | 0.161 | 0.042 |
| **Suc** | **Otu00025** | Bacteroidales BS11 gut group ge | 1.132 | 0.569 | **Otu00022** | Lachnospiraceae XPB1014 group | 0.810 | 0.251 |
|  | **Otu00040** | Bacteroidales RF16 group ge | 1.093 | 0.212 | **Otu00045** | *Prevotella* 1 | 0.302 | 0.220 |
|  | **Otu00069** | *Prevotella* 1 | 0.596 | 0.233 | **Otu00145** | Lachnospiraceae unclassified | 0.258 | 0.133 |
|  | **Otu00180** | Ruminococcaceae UCG-002 | 0.274 | 0.073 | **Otu00124** | Ruminococcaceae NK4A214 group | 0.133 | 0.036 |
|  | **Otu00242** | Bacteroidales S24-7 group ge | 0.085 | 0.043 | **Otu00157** | *Anaerotruncus* | 0.130 | 0.016 |
| **Val** | **Otu00098** | Prevotellaceae UCG-003 | 0.276 | 0.094 | **Otu00026** | Lachnospiraceae NK3A20 group | 0.694 | 0.183 |
|  | **Otu00209** | Rikenellaceae RC9 gut group | 0.228 | 0.075 | **Otu00098** | Prevotellaceae UCG-003 | 0.396 | 0.191 |
|  | **Otu00053** | *Treponema* 2 | 0.197 | 0.032 | **Otu00081** | Lachnospiraceae NK3A20 group | 0.299 | 0.057 |
|  | **Otu00093** | *Prevotella* 1 | 0.162 | 0.060 | **Otu00139** | Ruminococcaceae NK4A214 group | 0.249 | 0.141 |
|  | **Otu00236** | Victivallales vadinBE97 ge | 0.133 | 0.017 | **Otu00134** | Clostridiales Family XIII AD3011 group | 0.145 | 0.042 |
| **Isoval** | **Otu00021** | Christensenellaceae R-7 group | 0.616 | 0.311 | **Otu00075** | Christensenellaceae R-7 group | 0.604 | 0.452 |
|  | **Otu00067** | *Mogibacterium* | 0.391 | 0.138 | **Otu00050** | *Prevotella* 1 | 0.358 | 0.106 |
|  | **Otu00188** | Clostridiales Family XIII AD3011 group | 0.208 | 0.079 | **Otu00048** | Bacteroidales BS11 gut group ge | 0.293 | 0.043 |
|  | **Otu00201** | Verrucomicrobia WCHB1-41 ge | 0.134 | 0.023 | **Otu00211** | Ruminococcaceae unclassified | 0.139 | 0.034 |
|  | **Otu00102** | Rikenellaceae RC9 gut group | 0.132 | 0.030 | **Otu00405** | Ruminococcaceae unclassified | 0.066 | 0.014 |
| **Ace/Prop** | **Otu00156** | Ruminococcaceae UCG-005 | 0.204 | 0.086 | **Otu00070** | Ruminococcaceae NK4A214 group | 0.306 | 0.058 |
|  | **Otu00150** | Ruminococcaceae UCG-005 | 0.181 | 0.033 | **Otu00447** | Bacteroidales S24-7 group ge | 0.060 | 0.023 |
|  | **Otu00119** | Anaerolineaceae uncultured | 0.120 | 0.022 | **Otu00371** | *Treponema* 2 | 0.058 | 0.039 |
|  | **Otu00274** | Prevotellaceae UCG-003 | 0.084 | 0.014 | **Otu00342** | Chloroflexi unclassified | 0.057 | 0.018 |
|  | **Otu00426** | Rikenellaceae RC9 gut group | 0.068 | 0.025 | **Otu00319** | Ruminococcaceae UCG-010 | 0.055 | 0.016 |
| **VFAt** | **Otu00015** | Rikenellaceae RC9 gut group | 0.615 | 0.147 | **Otu00203** | Bacteroidales S24-7 group ge | 0.083 | 0.027 |
|  | **Otu00048** | Bacteroidales BS11 gut group ge | 0.416 | 0.065 | **Otu00316** | Anaerolineaceae unclassified | 0.077 | 0.010 |
|  | **Otu00018** | *Prevotella* 1 | 0.269 | 0.123 | **Otu00295** | *Prevotella* 1 | 0.073 | 0.028 |
|  | **Otu00066** | *Phocaeicola* | 0.240 | 0.043 | **Otu00284** | Christensenellaceae R-7 group | 0.068 | 0.011 |
|  | **Otu00157** | *Anaerotruncus* | 0.129 | 0.015 | **Otu00379** | Lachnospiraceae XPB1014 group | 0.066 | 0.013 |
| **pH** | **Otu00057** | Bacteroidales BS11 gut group ge | 0.357 | 0.155 | **Otu00012** | *Prevotella* 1 | 0.928 | 0.188 |
|  | **Otu00033** | Lachnospiraceae unclassified | 0.245 | 0.081 | **Otu00033** | Lachnospiraceae unclassified | 0.656 | 0.166 |
|  | **Otu00153** | *Saccharofermentans* | 0.178 | 0.114 | **Otu00095** | Rikenellaceae RC9 gut group | 0.392 | 0.049 |
|  | **Otu00197** | Rikenellaceae RC9 gut group | 0.119 | 0.054 | **Otu00163** | Lachnospiraceae probable genus 10 | 0.289 | 0.136 |
|  | **Otu00099** | *Prevotella* 1 | 0.088 | 0.023 | **Otu00155** | *Ruminococcus* 1 | 0.232 | 0.125 |
| **NH** | **Otu00036** | Rikenellaceae U29-B03 | 0.574 | 0.259 | **Otu00024** | *Succiniclasticum* | 0.754 | 0.128 |
|  | **Otu00140** | Rikenellaceae RC9 gut group | 0.291 | 0.099 | **Otu00098** | Prevotellaceae UCG-003 | 0.396 | 0.191 |
|  | **Otu00098** | Prevotellaceae UCG-003 | 0.276 | 0.094 | **Otu00018** | *Prevotella* 1 | 0.340 | 0.140 |
|  | **Otu00018** | *Prevotella* 1 | 0.269 | 0.123 | **Otu00139** | Ruminococcaceae NK4A214 group | 0.249 | 0.141 |
|  | **Otu00053** | *Treponema* 2 | 0.197 | 0.032 | **Otu00125** | Ruminococcaceae NK4A214 group | 0.134 | 0.014 |

^a^Fermentation parameters: molar proportion of acetic, Ace; propionic, Prop; succinic, Suc; butyric, But; isobutyric, Isobut; valeric, val; and isovaleric acids, Isoval; acetate-to-propionate ratio, Ace/Prop; total concentration of these volatile fats acids, VFAt; ammonia concentration, NH_3_; and pH

^b^Taxonomy for each OTU is given at the highest classifiable level

^c^Standard error of mean (SEM)

**Supplementary Table S8** Relative abundance of the most abundant bacterial OTUs showing negative correlation with ruminal fermentation parameters^a^ in p-RFI steers

| **Parameter** | **Liquid** | | | | **Solid** | | | |
| --- | --- | --- | --- | --- | --- | --- | --- | --- |
|  | **OTU** | **Taxonomy^b^** | **Relative abundance (%)** | **SEM^c^** | **OTU** | **Taxonomy^b^** | **Relative abundance (%)** | **SEM^c^** |
| **Ace** | **Otu00044** | Christensenellaceae R-7 group | 0.629 | 0.550 | **Otu00019** | Prevotellaceae NK3B31 group | 1.023 | 0.805 |
|  | **Otu00019** | Prevotellaceae NK3B31 group | 0.504 | 0.383 | **Otu00234** | Ruminococcaceae ge | 0.069 | 0.019 |
|  | **Otu00048** | Bacteroidales BS11 gut group ge | 0.448 | 0.068 | **Otu00220** | Bacteroidales BS11 gut group ge | 0.061 | 0.035 |
|  | **Otu00035** | *Mogibacterium* | 0.415 | 0.152 | **Otu00529** | Clostridiales unclassified | 0.054 | 0.013 |
|  | **Otu00181** | Clostridiales Family XIII unclassified | 0.143 | 0.034 | **Otu00519** | Clostridiales Family XIII AD3011 group | 0.049 | 0.013 |
| **Prop** | **Otu00032** | Rikenellaceae RC9 gut group | 0.868 | 0.235 | **Otu00015** | Rikenellaceae RC9 gut group | 1.026 | 0.299 |
|  | **Otu00064** | *Prevotella* 1 | 0.468 | 0.198 | **Otu00011** | *Prevotella* 1 | 0.506 | 0.094 |
|  | **Otu00015** | Rikenellaceae RC9 gut group | 0.464 | 0.151 | **Otu00095** | Rikenellaceae RC9 gut group | 0.407 | 0.071 |
|  | **Otu00207** | *Spirochaeta* 2 | 0.114 | 0.019 | **Otu00064** | *Prevotella* 1 | 0.350 | 0.107 |
|  | **Otu00105** | Lachnospiraceae FCS020 group | 0.083 | 0.022 | **Otu00072** | Ruminococcaceae ge | 0.192 | 0.044 |
| **But** | **Otu00057** | Bacteroidales BS11 gut group ge | 0.461 | 0.181 | **Otu00130** | Prevotellaceae YAB2003 group | 0.307 | 0.045 |
|  | **Otu00148** | Bacteroidales S24-7 group ge | 0.118 | 0.050 | **Otu00099** | *Prevotella* 1 | 0.275 | 0.100 |
|  | **Otu00170** | Rikenellaceae RC9 gut group | 0.100 | 0.017 | **Otu00133** | *Saccharofermentans* | 0.214 | 0.042 |
|  | **Otu00241** | Bacteroidales S24-7 group ge | 0.078 | 0.018 | **Otu00231** | *Papillibacter* | 0.163 | 0.062 |
|  | **Otu00362** | Verrucomicrobia WCHB1-41 ge | 0.068 | 0.018 | **Otu00106** | Bacteroidales BS11 gut group ge | 0.128 | 0.039 |
| **Isobut** | **Otu00156** | Ruminococcaceae UCG-005 | 0.148 | 0.049 | **Otu00033** | Lachnospiraceae unclassified | 0.753 | 0.143 |
|  | **Otu00173** | Ruminococcaceae ge | 0.121 | 0.043 | **Otu00087** | Lachnospiraceae XPB1014 group | 0.394 | 0.049 |
|  | **Otu00198** | Planctomycetaceae p-1088-a5 gut group | 0.097 | 0.026 | **Otu00210** | Bacteroidales BS11 gut group ge | 0.077 | 0.059 |
|  | **Otu00400** | Clostridiales vadinBB60 group ge | 0.082 | 0.019 | **Otu00327** | *Treponema* 2 | 0.076 | 0.020 |
|  | **Otu00258** | Prevotellaceae UCG-004 | 0.082 | 0.043 | **Otu00414** | *Treponema* 2 | 0.049 | 0.007 |
| **Suc** | **Otu00012** | *Prevotella* 1 | 1.024 | 0.256 | **Otu00030** | *Prevotella* 1 | 0.697 | 0.267 |
|  | **Otu00061** | Bacteroidales S24-7 group ge | 0.464 | 0.255 | **Otu00012** | *Prevotella* 1 | 0.679 | 0.121 |
|  | **Otu00050** | *Prevotella* 1 | 0.383 | 0.106 | **Otu00055** | Christensenellaceae R-7 group | 0.411 | 0.076 |
|  | **Otu00055** | Christensenellaceae R-7 group | 0.337 | 0.046 | **Otu00271** | *Ruminococcus* 1 | 0.069 | 0.012 |
|  | **Otu00261** | Lachnospiraceae UCG-008 | 0.104 | 0.024 | **Otu00422** | Bacteroidales UCG-001 ge | 0.048 | 0.016 |
| **Val** | **Otu00058** | Ruminococcaceae UCG-005 | 0.366 | 0.076 | **Otu00014** | *Saccharofermentans* | 1.499 | 0.177 |
|  | **Otu00232** | *Prevotella* 1 | 0.133 | 0.067 | **Otu00137** | Lachnospiraceae UCG-009 | 0.286 | 0.032 |
|  | **Otu00236** | Victivallales vadinBE97 ge | 0.112 | 0.017 | **Otu00107** | Prevotellaceae NK3B31 group | 0.211 | 0.182 |
|  | **Otu00389** | Bacteroidales BS11 gut group ge | 0.080 | 0.059 | **Otu00405** | Ruminococcaceae unclassified | 0.077 | 0.010 |
|  | **Otu00126** | *Ruminococcus* 1 | 0.077 | 0.015 | **Otu00295** | *Prevotella* 1 | 0.063 | 0.011 |
| **Isoval** | **Otu00156** | Ruminococcaceae UCG-005 | 0.148 | 0.049 | **Otu00053** | *Treponema* 2 | 0.509 | 0.109 |
|  | **Otu00263** | *Ruminococcus* 1 | 0.096 | 0.044 | **Otu00087** | Lachnospiraceae XPB1014 group | 0.394 | 0.049 |
|  | **Otu00635** | *Howardella* | 0.038 | 0.007 | **Otu00068** | Lachnospiraceae ge | 0.360 | 0.059 |
|  | **Otu00617** | Spirochaetaceae unclassified | 0.021 | 0.010 | **Otu00114** | Lachnospiraceae unclassified | 0.216 | 0.016 |
|  | **Otu00612** | Prevotellaceae UCG-001 | 0.020 | 0.009 | **Otu00177** | *Fibrobacter* | 0.176 | 0.043 |
| **Ace/Prop** | **Otu00003** | Rikenellaceae RC9 gut group | 1.951 | 0.399 | **Otu00003** | Rikenellaceae RC9 gut group | 1.752 | 0.328 |
|  | **Otu00044** | Christensenellaceae R-7 group | 0.629 | 0.550 | **Otu00008** | *Saccharofermentans* | 1.528 | 0.259 |
|  | **Otu00048** | Bacteroidales BS11 gut group ge | 0.448 | 0.068 | **Otu00018** | *Prevotella* 1 | 0.767 | 0.530 |
|  | **Otu00198** | Planctomycetaceae p-1088-a5 gut group | 0.097 | 0.026 | **Otu00078** | Lachnospiraceae ge | 0.331 | 0.065 |
|  | **Otu00214** | *Saccharofermentans* | 0.094 | 0.026 | **Otu00066** | *Phocaeicola* | 0.293 | 0.101 |
| **VFAt** | **Otu00059** | Ruminococcaceae UCG-005 | 0.325 | 0.092 | **Otu00015** | Rikenellaceae RC9 gut group | 1.026 | 0.299 |
|  | **Otu00096** | *Prevotella* 1 | 0.288 | 0.121 | **Otu00023** | *Papillibacter* | 0.955 | 0.062 |
|  | **Otu00143** | Prevotellaceae UCG-001 | 0.230 | 0.056 | **Otu00031** | *Prevotella* 1 | 0.619 | 0.307 |
|  | **Otu00091** | Ruminococcaceae NK4A214 group | 0.185 | 0.072 | **Otu00042** | *Prevotella* 1 | 0.334 | 0.158 |
|  | **Otu00192** | Ruminococcaceae ge | 0.162 | 0.047 | **Otu00096** | *Prevotella* 1 | 0.309 | 0.131 |
| **pH** | **Otu00018** | *Prevotella* 1 | 0.795 | 0.611 | **Otu00003** | Rikenellaceae RC9 gut group | 1.752 | 0.328 |
|  | **Otu00044** | Christensenellaceae R-7 group | 0.629 | 0.550 | **Otu00052** | Clostridiales unclassified | 0.394 | 0.108 |
|  | **Otu00048** | Bacteroidales BS11 gut group ge | 0.448 | 0.068 | **Otu00048** | Bacteroidales BS11 gut group ge | 0.350 | 0.061 |
|  | **Otu00035** | *Mogibacterium* | 0.415 | 0.152 | **Otu00258** | Prevotellaceae UCG-004 | 0.104 | 0.045 |
|  | **Otu00234** | Ruminococcaceae ge | 0.089 | 0.023 | **Otu00234** | Ruminococcaceae ge | 0.069 | 0.019 |
| **NH** | **Otu00072** | Ruminococcaceae ge | 0.343 | 0.084 | **Otu00028** | Rikenellaceae RC9 gut group | 0.735 | 0.126 |
|  | **Otu00201** | Verrucomicrobia WCHB1-41 ge | 0.114 | 0.011 | **Otu00074** | Lachnospiraceae XPB1014 group | 0.358 | 0.068 |
|  | **Otu00381** | Ruminococcaceae UCG-001 | 0.077 | 0.042 | **Otu00114** | Lachnospiraceae unclassified | 0.216 | 0.016 |
|  | **Otu00504** | Rikenellaceae RC9 gut group | 0.037 | 0.016 | **Otu00072** | Ruminococcaceae ge | 0.192 | 0.044 |
|  | **Otu00413** | Mollicutes RF9 ge | 0.032 | 0.010 | **Otu00149** | Lachnospiraceae XPB1014 group | 0.167 | 0.031 |

^a^Fermentation parameters: molar proportion of acetic, Ace; propionic, Prop; succinic, Suc; butyric, But; isobutyric, Isobut; valeric, val; and isovaleric acids, Isoval; acetate-to-propionate ratio, Ace/Prop; total concentration of these volatile fats acids, VFAt; ammonia concentration, NH_3_; and pH

^b^Taxonomy for each OTU is given at the highest classifiable level

^c^Standard error of mean (SEM)

**Supplementary Table S9** Relative abundance of the most abundant bacterial OTUs showing negative correlation with ruminal fermentation parameters^a^ in n-RFI steers

| **Parameter** | **Liquid** | | | | **Solid** | | | |  |
| --- | --- | --- | --- | --- | --- | --- | --- | --- | --- |
|  | **OTU** | **Taxonomy^b^** | **Relative abundance (%)** | **SEM^c^** | **OTU** | **Taxonomy^b^** | **Relative abundance (%)** | **SEM^c^** | |
| **Ace** | **Otu00036** | Rikenellaceae U29-B03 | 0.574 | 0.259 | **Otu00026** | Lachnospiraceae NK3A20 group | 0.694 | 0.183 | |
|  | **Otu00111** | *Prevotella* 1 | 0.298 | 0.113 | **Otu00226** | *Prevotella* 1 | 0.147 | 0.019 | |
|  | **Otu00190** | Rikenellaceae RC9 gut group | 0.117 | 0.029 | **Otu00118** | Prevotellaceae UCG-003 | 0.130 | 0.017 | |
|  | **Otu00220** | Bacteroidales BS11 gut group ge | 0.084 | 0.031 | **Otu00412** | Anaerolineaceae uncultured | 0.082 | 0.058 | |
|  | **Otu00699** | Verrucomicrobia WCHB1-41 ge | 0.040 | 0.015 | **Otu00354** | Clostridiales Family XIII ge | 0.053 | 0.016 | |
| **Prop** | **Otu00119** | Anaerolineaceae uncultured | 0.120 | 0.022 | **Otu00063** | *Prevotella* 1 | 0.072 | 0.018 | |
|  | **Otu00367** | Chloroflexi unclassified | 0.105 | 0.050 | **Otu00359** | *Fretibacterium* | 0.070 | 0.013 | |
|  | **Otu00274** | Prevotellaceae UCG-003 | 0.084 | 0.014 | **Otu00319** | Ruminococcaceae UCG-010 | 0.055 | 0.016 | |
|  | **Otu00426** | Rikenellaceae RC9 gut group | 0.068 | 0.025 | **Otu00407** | Anaerolineaceae unclassified | 0.050 | 0.014 | |
|  | **Otu00575** | Ruminococcaceae unclassified | 0.062 | 0.027 | **Otu00040** | Bacteroidales RF16 group ge | 0.050 | 0.008 | |
| **But** | **Otu00078** | Lachnospiraceae ge | 0.178 | 0.031 | **Otu00129** | Lachnospiraceae unclassified | 0.279 | 0.121 | |
|  | **Otu00122** | Rikenellaceae RC9 gut group | 0.165 | 0.039 | **Otu00227** | Prevotellaceae unclassified | 0.173 | 0.033 | |
|  | **Otu00129** | Lachnospiraceae unclassified | 0.154 | 0.060 | **Otu00256** | Prevotellaceae NK3B31 group | 0.171 | 0.081 | |
|  | **Otu00165** | Lachnospiraceae XPB1014 group | 0.091 | 0.017 | **Otu00165** | Lachnospiraceae XPB1014 group | 0.160 | 0.033 | |
|  | **Otu00088** | *Fibrobacter* | 0.052 | 0.032 | **Otu00162** | Bacteroidales BS11 gut group ge | 0.111 | 0.075 | |
| **Isobut** | **Otu00098** | Prevotellaceae UCG-003 | 0.276 | 0.094 | **Otu00098** | Prevotellaceae UCG-003 | 0.396 | 0.191 | |
|  | **Otu00053** | *Treponema* 2 | 0.197 | 0.032 | **Otu00018** | *Prevotella* 1 | 0.340 | 0.140 | |
|  | **Otu00109** | Prevotellaceae UCG-004 | 0.137 | 0.056 | **Otu00082** | *Moryella* | 0.324 | 0.048 | |
|  | **Otu00152** | *Anaerovorax* | 0.136 | 0.021 | **Otu00068** | Lachnospiraceae ge | 0.254 | 0.037 | |
|  | **Otu00167** | Rikenellaceae RC9 gut group | 0.122 | 0.035 | **Otu00187** | *Acetitomaculum* | 0.194 | 0.064 | |
| **Suc** | **Otu00084** | Prevotellaceae UCG-001 | 0.345 | 0.098 | **Otu00110** | Lachnospiraceae NK4A136 group | 0.438 | 0.199 | |
|  | **Otu00116** | Lachnospiraceae ND3007 group | 0.316 | 0.109 | **Otu00020** | *Prevotella* 1 | 0.373 | 0.114 | |
|  | **Otu00018** | *Prevotella* 1 | 0.269 | 0.123 | **Otu00149** | Lachnospiraceae XPB1014 group | 0.282 | 0.037 | |
|  | **Otu00020** | *Prevotella* 1 | 0.267 | 0.095 | **Otu00104** | Ruminococcaceae NK4A214 group | 0.164 | 0.042 | |
|  | **Otu00179** | Christensenellaceae R-7 group | 0.120 | 0.079 | **Otu00208** | Lachnospiraceae unclassified | 0.140 | 0.025 | |
| **Val** | **Otu00001** | Christensenellaceae R-7 group | 5.419 | 0.980 | **Otu00033** | Lachnospiraceae unclassified | 0.656 | 0.166 | |
|  | **Otu00007** | Ruminococcaceae NK4A214 group | 1.647 | 0.214 | **Otu00095** | Rikenellaceae RC9 gut group | 0.392 | 0.049 | |
|  | **Otu00035** | *Mogibacterium* | 0.356 | 0.079 | **Otu00122** | Rikenellaceae RC9 gut group | 0.172 | 0.042 | |
|  | **Otu00156** | Ruminococcaceae UCG-005 | 0.204 | 0.086 | **Otu00240** | *Fibrobacter* | 0.123 | 0.065 | |
|  | **Otu00124** | Ruminococcaceae NK4A214 group | 0.186 | 0.067 | **Otu00330** | *Treponema* 2 | 0.064 | 0.015 | |
| **Isoval** | **Otu00059** | Ruminococcaceae UCG-005 | 0.615 | 0.237 | **Otu00099** | *Prevotella* 1 | 0.228 | 0.066 | |
|  | **Otu00255** | Clostridiales Family XIII ge | 0.207 | 0.078 | **Otu00183** | *Ruminococcus* 1 | 0.221 | 0.102 | |
|  | **Otu00314** | Bacteroidales S24-7 group ge | 0.139 | 0.054 | **Otu00166** | Peptococcaceae uncultured | 0.191 | 0.045 | |
|  | **Otu00469** | Ruminococcaceae unclassified | 0.062 | 0.021 | **Otu00096** | *Prevotella* 1 | 0.118 | 0.053 | |
|  | **Otu00134** | Clostridiales Family XIII AD3011 group | 0.061 | 0.018 | **Otu00293** | *Succiniclasticum* | 0.092 | 0.026 | |
| **Ace/Prop** | **Otu00326** | Prevotellaceae UCG-003 | 0.182 | 0.094 | **Otu00109** | Prevotellaceae UCG-004 | 0.168 | 0.071 | |
|  | **Otu00317** | Rikenellaceae RC9 gut group | 0.083 | 0.027 | **Otu00215** | Lachnospiraceae UCG-002 | 0.113 | 0.016 | |
|  | **Otu00297** | Prevotellaceae UCG-001 | 0.063 | 0.016 | **Otu00310** | Clostridiales Family XIII AD3011 group | 0.093 | 0.037 | |
|  | **Otu00347** | Ruminococcaceae UCG-002 | 0.058 | 0.010 | **Otu00375** | Lachnospiraceae unclassified | 0.075 | 0.016 | |
|  | **Otu00160** | Ruminococcus 1 | 0.056 | 0.019 | **Otu00347** | Ruminococcaceae UCG-002 | 0.068 | 0.018 | |
| **VFAt** | **Otu00101** | Veillonellaceae UCG-001 | 0.202 | 0.017 | **Otu00011** | *Prevotella* 1 | 0.311 | 0.051 | |
|  | **Otu00107** | Prevotellaceae NK3B31 group | 0.150 | 0.044 | **Otu00072** | Ruminococcaceae ge | 0.161 | 0.042 | |
|  | **Otu00310** | Clostridiales Family XIII AD3011 group | 0.079 | 0.016 | **Otu00153** | *Saccharofermentans* | 0.142 | 0.067 | |
|  | **Otu00345** | *Senegalimassilia* | 0.047 | 0.014 | **Otu00135** | Bacteroidales S24-7 group ge | 0.141 | 0.075 | |
|  | **Otu00374** | *Senegalimassilia* | 0.039 | 0.007 | **Otu00221** | Lachnospiraceae XPB1014 group | 0.086 | 0.027 | |
| **pH** | **Otu00140** | Rikenellaceae RC9 gut group | 0.291 | 0.099 | **Otu00018** | *Prevotella* 1 | 0.340 | 0.140 | |
|  | **Otu00098** | Prevotellaceae UCG-003 | 0.276 | 0.094 | **Otu00068** | Lachnospiraceae ge | 0.254 | 0.037 | |
|  | **Otu00053** | *Treponema* 2 | 0.197 | 0.032 | **Otu00139** | Ruminococcaceae NK4A214 group | 0.249 | 0.141 | |
|  | **Otu00152** | *Anaerovorax* | 0.136 | 0.021 | **Otu00114** | Lachnospiraceae unclassified | 0.211 | 0.023 | |
|  | **Otu00167** | Rikenellaceae RC9 gut group | 0.122 | 0.035 | **Otu00179** | Christensenellaceae R-7 group | 0.187 | 0.140 | |
| **NH** | **Otu00001** | Christensenellaceae R-7 group | 5.419 | 0.980 | **Otu00033** | Lachnospiraceae unclassified | 0.656 | 0.166 | |
|  | **Otu00035** | *Mogibacterium* | 0.356 | 0.079 | **Otu00042** | *Prevotella* 1 | 0.405 | 0.145 | |
|  | **Otu00042** | *Prevotella* 1 | 0.334 | 0.091 | **Otu00095** | Rikenellaceae RC9 gut group | 0.392 | 0.049 | |
|  | **Otu00101** | Veillonellaceae UCG-001 | 0.202 | 0.017 | **Otu00155** | *Ruminococcus* 1 | 0.232 | 0.125 | |
|  | **Otu00119** | Anaerolineaceae uncultured | 0.120 | 0.022 | **Otu00251** | Bacteroidetes BD2-2 ge | 0.118 | 0.023 | |

^a^Fermentation parameters: molar proportion of acetic, Ace; propionic, Prop; succinic, Suc; butyric, But; isobutyric, Isobut; valeric, val; and isovaleric acids, Isoval; acetate-to-propionate ratio, Ace/Prop; total concentration of these volatile fats acids, VFAt; ammonia concentration, NH_3_; and pH

^b^Taxonomy for each OTU is given at the highest classifiable level

^c^Standard error of mean (SEM)
